# Isolation, characterization and comparison of Atlantic and Chinook salmon growth hormone 1 and 2

**DOI:** 10.1186/1471-2164-9-522

**Published:** 2008-11-03

**Authors:** Kristian R von Schalburg, Ryosuke Yazawa, Johan de Boer, Krzysztof P Lubieniecki, Benjamin Goh, Christopher A Straub, Marianne R Beetz-Sargent, Adrienne Robb, William S Davidson, Robert H Devlin, Ben F Koop

**Affiliations:** 1 Centre for Biomedical Research, University of Victoria, Victoria, British Columbia, V8W 3N5,Canada; 2 Department of Molecular Biology and Biochemistry, Simon Fraser University, Burnaby, British Columbia, V5A 1S6, Canada,; 3 Fisheries and Oceans Canada, 4160 Marine Drive, West Vancouver, British Columbia, V7V 1N6, Canada

## Abstract

**Background:**

Growth hormone (GH) is an important regulator of skeletal growth, as well as other adapted processes in salmonids. The GH gene (*gh*) in salmonids is represented by duplicated, non-allelic isoforms designated as *gh1 *and *gh2*. We have isolated and characterized *gh*-containing bacterial artificial chromosomes (BACs) of both Atlantic and Chinook salmon (*Salmo salar *and *Oncorhynchus tshawytscha*) in order to further elucidate our understanding of the conservation and regulation of these loci.

**Results:**

BACs containing *gh1 *and *gh2 *from both Atlantic and Chinook salmon were assembled, annotated, and compared to each other in their coding, intronic, regulatory, and flanking regions. These BACs also contain the genes for skeletal muscle sodium channel oriented in the same direction. The sequences of the genes for interferon alpha-1, myosin alkali light chain and microtubule associated protein Tau were also identified, and found in opposite orientations relative to *gh1 *and *gh2*. Viability of each of these genes was examined by PCR. We show that transposon insertions have occurred differently in the promoters of *gh*, within and between each species. Other differences within the promoters and intronic and 3'-flanking regions of the four *gh *genes provide evidence that they have distinct regulatory modes and possibly act to function differently and/or during different times of salmonid development.

**Conclusion:**

A core proximal promoter for transcription of both *gh1 *and *gh2 *is conserved between the two species of salmon. Nevertheless, transposon integration and regulatory element differences do exist between the promoters of *gh1 *and *gh2*. Additionally, organization of transposon families into the BACs containing *gh1 *and for the BACs containing *gh2*, are very similar within orthologous regions, but much less clear conservation is apparent in comparisons between the *gh1*- and *gh2*-containing paralogous BACs for the two fish species. This is consistent with the hypothesis that a burst of transposition activity occurred during the speciation events which led to Atlantic and Pacific salmon. The Chinook and other *Oncorhynchus *GH1s are strikingly different in comparison to the other GHs and this change is not apparent in the surrounding non-coding sequences.

## Background

Salmonids are used as models for studies in environmental toxicology, physiology, comparative immunology, growth, gametogenesis, olfaction and osmoregulation [1]. Although considerable knowledge of the basic biology of salmonids exists [1-3], the characterization of salmonid genomes will better enable scientific decisions on the conservation and enhancement of wild stocks, improve knowledge of fish health and increase the commercial viability of aquaculture. Sequencing of salmonid genomes also permits investigators to study fundamental questions concerning genome evolution, genome duplication and rearrangements, repeat-rich structures, transposon activity, gene silencing, and the re-establishment of a more stable diploid genome from a pseudotetraploid state.

Growth hormone (GH) plays a very important role in many regulatory, metabolic and developmental processes in various vertebrate tissues [4]. In salmonids, GH is the principle stimulator of skeletal growth and plays a key role in lipid mobilization, protein synthesis and feeding behaviour [5]. In various fishes, GH also manifests some functions not found in all vertebrates, such as activities that influence sexual maturation and saltwater adaptation [6,7]. The GH gene (*gh*) in salmonids is represented by duplicated, non-allelic isoforms designated as *gh1 *and *gh2 *[8], which diverged at least 30 million years (MY) ago [9].

In vertebrates, the major source of GH is the somatotrophic cells in the pituitary, from which it is secreted into and borne by the plasma to act on receptors throughout the organism [5,10]. Splice variants have also been isolated from extrapituitary tissue in humans (placenta, testis, blood mononuclear cells) and chicken (eye, heart) [[11] and refs. therein]. In trout, *gh *transcripts have been detected in the pituitary, liver, head kidney, spleen, thymus, intestine and leukocytes and gut [12,13]. Improved understanding of the chromosomal environment in which *gh *genes reside in salmonids will assist our understanding of the transcriptional controls regulating these genes. By analyzing two paralogous genes (e.g. *gh1 *and *gh2*) and doing so in two species (which have been separated for approximately 20 MY; [9]), identification of common conserved regions with presumed broad functional importance can be achieved. Further, regions which are found to be conserved only between species in one paralogue type may be important for differential regulatory control between the paralogues, while differences between species within a single paralogue type may help identify regions not important to regulation. Regions which are conserved between paralogues in only one species are candidates for regions which have undergone gene conversion subsequent to divergence between the two species. While gene conversion does not appear to have occurred at salmonid GH loci when examined at the gene level [9], an analysis of gene conversion has not been examined at larger scales now possible with genomic analyses. Understanding these sequence relationships has important practical ramifications since complete genomic resources are being developed for Atlantic salmon. It is important to determine the degree of conservation of this information with other important salmonid species such as Chinook salmon for extrapolation of emerging genomic information from one species to another.

Bacterial artificial chromosomes (BACs) containing *gh1 *and *gh2 *from both Atlantic and Chinook salmon were assembled, annotated, and compared to each other in their coding, intronic, regulatory, and flanking regions. A core proximal promoter for transcription of both *gh1 *and *gh2 *is conserved between the two species of salmon. A 1600 bp insertion of a Tc1-like DNA transposon sequence was found within the promoter region of both *gh2 *genes, but not in the promoter of the *gh1 *genes. Furthermore, a Polinton-1 transposon is inserted in only the promoter for Chinook salmon GH1. Other differences within the promoters and intronic and 3'-flanking regions of the four genes provide evidence that supports the notion that they are regulated differently and thus may possess different functions. Intriguingly, Chinook salmon GH1 has undergone more than twice as many changes than any of the other GHs; changes not reflected in the surrounding non-coding DNA.

## Results

Confirmation that the Atlantic salmon BACs we isolated were *gh*-containing BACs was performed by comparing *Hind*III-digested BAC fragment profiles to profiles on the internet Contig Explorer version 3.4 (iCE 3.4) database [14]. The gene for the skeletal muscle sodium channel (*scn*) was also identified by PCR for each isolated BAC, suggesting that each BAC contained the 5'-region upstream of each *gh *gene. *gh *type *(gh1 *vs. *gh2*) was determined for BACs in both Atlantic and Chinook salmon by paralogue-specific PCR.

### BAC and GH comparisons

**Figure 1:**
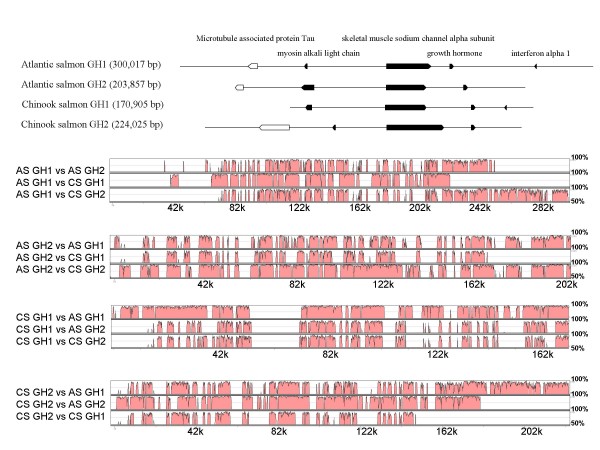
**A) The genomic organization of growth hormone 1 and 2 gene loci in Atlantic and Chinook salmon**. Approximate sizes of the sequenced bacterial artificial chromosomes are indicated in parentheses. Growth hormone 1 and 2 genes and their flanking genes are represented by boxes with transcriptional directions indicated by arrowheads. The introns for the genes are not shown. Pseudogenes are indicated by open boxes. **B) A comparison of Atlantic salmon and Chinook salmon *gh1 *and *gh2 *loci shown in VISTA plots**. Regions of high similarity (50 to 100%; vertical axis) are separated by gaps that represent repeat sequences, deletions and insertions. Orthologous comparisons (AS *gh1 *and CS *gh1*, and AS *gh2 *and CS *gh2*) appear more similar than paralogous comparisons (*gh1 *and *gh2*).

Atlantic salmon (AS) and Chinook salmon (CS)*gh *loci were analyzed using DIGIT [15], which identified the presence, location, and direction of putative genes on each BAC. Each of these putative genes were assessed by BLASTX [16] to protein databases. BACs containing the *gh *genes also bore the genes for *scn *oriented in the same direction (Figure 1A). The sequences of the genes for interferon alpha-1 (*ifna1*), myosin alkali light chain (*mlc*) and microtubule associated protein Tau (*mapt*) were also identified, and found in opposite orientations relative to *gh1 *and *gh2 *(Figure 1A). The Chinook salmon *gh1 *and *gh2*-containing BACs are similarly organized.

An overall comparison of Atlantic and Chinook salmon *gh1 *and *gh2 *loci are shown in VISTA plots [17] (Figure 1B). This figure shows many regions of high similarity separated by gaps that represent repeat sequences, deletions and insertions. Visually orthologous comparisons (AS *gh1 *and CS *gh1*, and AS *gh2 *and CS *gh2*) appear more similar than paralogous comparisons (*gh1 *and *gh2*).

Using RepeatMasker [18] and a new salmonid repeat database [19], 30% of the CS *gh1*, 28% of the CS *gh2*, 33% of AS *gh1 *and 35% of AS *gh2 *are repeated elements or simple repeats (approximately 50% of the total repeats are 1.6 kb Tc1-like transposons). With repeats removed from the genomic sequences, paralogous comparisons showed that Atlantic salmon *gh1 *and *gh2 *have 89.5% identity over 46,425 bp, and Chinook salmon *gh1 *and *gh2 *showed 87.9% identity over 52,782 bp. These alignments also revealed numerous insertions and deletions among these loci.

In comparisons of Atlantic and Chinook salmon BACs, *gh1 *showed 92.3% identity over 85,987 bp and *gh2 *showed 93.4% identity over 78,106 bp. Estimates of 93% identity between Atlantic salmon and Chinook salmon are similar to the 94% identity found between Atlantic salmon and rainbow trout (*Oncorhynchus mykiss*) over 125 kb [20].

**Figure 2:**
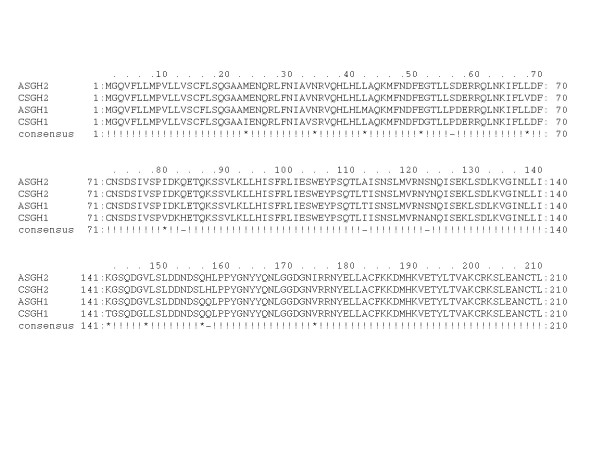
**Amino acid residue alignments of Atlantic and Chinook salmon GH1 and 2**. Identical residues designated by exclamation marks; asterisks denote strong residue similarity.

Comparisons of the 210 amino acid residues of GH show very strong similarity (Figure 2). Atlantic salmon GH1 and GH2 shows 97.1% identity, while Chinook salmon GH1 and GH2 shows 93.1% identity, and Atlantic salmon and Chinook salmon GH1 and GH2 shows 94.1% identity and 97.1% identity, respectively. An analysis of these relationships indicates that the CS GH1 has undergone more than twice as many changes than for any of the other GHs. This change in CS GH1 is not apparent in the surrounding non-coding sequences and indicates there may have been a shift of natural selection pressures on the coding region of the CS *gh1*.

### Gene annotation

The *gh *paralogs were fully annotated and the genes consist of 6 exons and 5 introns. All upstream positions we document are presented in relationship to the translation start site (in negative numbers) and, due to variations in insertions and deletions between the *gh *genes within their intronic regions, all downstream information is based on nucleotide (nt) numbering as presented in Additional file 1.

### GH promoter and intron analysis

A TATA box for all four *gh *genes is found at position -94 to -87 [see Additional file 1]. Each gene has an asymmetric cAMP-response element (CRE) (TGACG) at -217/-213 in the proximal promoter and a palindromic CRE at position 3563/3570 within intron 4, but the *gh2 *genes have single nt substitutions in the fourth base at this location [see Additional file 1]. There are at least four potential pituitary transcriptional activator-1 (Pit-1) binding elements (coordinates: -351/-338, -249/-237, -147/-134, and -119/-106) that are shared by each gene and two upstream, reverse-oriented Pit-1 binding motifs in only the *gh2 *promoters (TATGTTATTTAAAC). A retinoic acid receptor/retinoid × receptor (RAR/RXR) binding element is also located here at position -190 to -171. A potential estrogen response element (ERE) can be found in each promoter (GGCCAnnnTGACA), roughly 6,600 bp and 12,000 bp upstream of the transcription start site in both the *gh1 *and *gh2 *genes, respectively. Another potential ERE (GGTCAnnnnnTGAGC), found in only the *gh2 *promoters, is located about 6,200 bp and 10,200 bp upstream of the CS and AS *gh *genes, respectively.

Intron 3 of the *gh2 *genes are much shorter than the corresponding intron for the *gh1 *paralogs, partly accounted for by insertion of SSsp2201 microsatellites within them. Intron 5 of CS *gh1 *is at least 360 nts longer than for any of the other genes. Also, numerous polymorphic loci exist in intron 4 of the *gh *genes that have been previously characterized for salmonids, including AS and CS (coordinates: 2895/2917; 3060/3083; 3635/3673 and 3822/3865) [8]. The sequences from position 3060 to 3083 match identically [see Additional file 1], but we show that there is one nucleotide difference in the AS *gh1 *paralogue at position 3069. All of the other polymorphic loci match identically.

There are three polyadenylation (poly(A)) tail signal sequences (AATAAA) beginning at positions 5264, 5617 and 5717 [see Additional file 1]. All four *gh *genes have at least one poly(A) signal sequence, but only the *gh1 *genes potentially possess three canonical signal sequences.

### Transposon integration analysis

**Figure 3:**
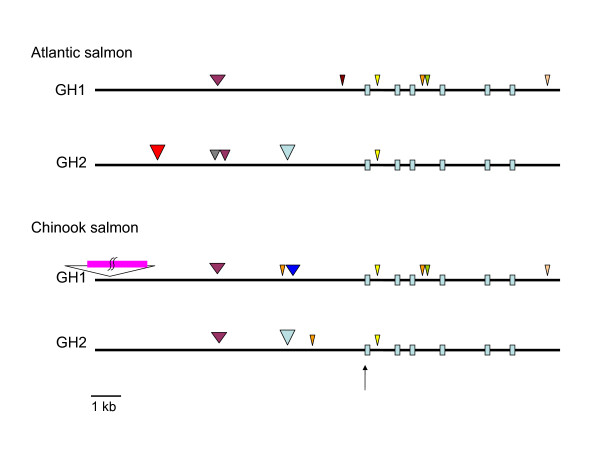
**Transposon integration in Atlantic and Chinook salmon *gh *promoter regions**. The insertion position of different transposon DNA sequences for each promoter are designated by coloured arrowheads and their corresponding family names. The arrow indicates the start of transcription, with approximately 7 kb shown for the promoter region, and 6 kb following the transcription start. Scale is approximate. Light blue boxes: exons 1 to 6. Purple arrowheads: DTSsa7 DNA transposon [Genbank:EF685960]; red arrowhead: DTSsa2 DNA transposon [Genbank:EF685955]; grey arrowhead: pTSsa1 DNA transposon [Genbank:EF685966]; light blue arrowheads: Tss DNA transposon [Genbank:L12207]; brown arrowheads: BHMS202 microsatellite [Genbank:AF256894]; dark blue arrowhead: SsaRT.3 LINE [unpublished]; orange arrowheads: HpaI SINE [Genbank:AY703447]; green arrowheads: Sssp2201 microsatellite [Genbank:AY081807]; yellow arrowheads: SaSN2b SINE [unpublished]; cream arrowheads: C43 sequence repeat [unpublished]; purple rectangle: palindrome-containing Polinton-like insert (20 kb total).

**Figure 4:**
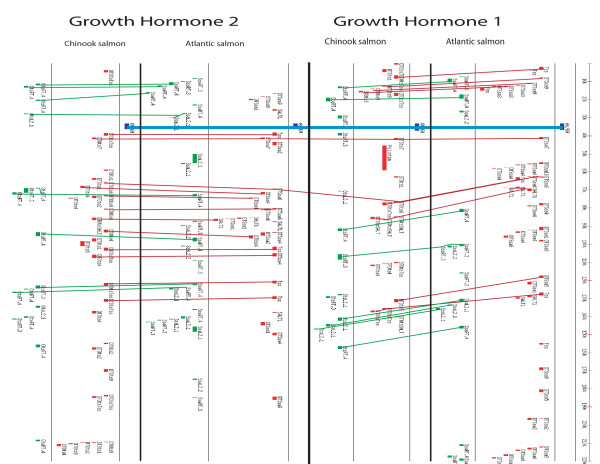
**The distribution of DNA transposons and LINE sequence elements in *gh1*- and *gh2*-containing BACs**. The growth hormone coding sequence (consisting of 6 exons) is aligned for the four BACs and connected by a light blue line. The conservation of the order of transposon families is indicated by lines, connecting corresponding sequences.

A Tc1-like DNA transposon sequence (Tss, [Genbank:L12207]) is inserted into the promoter region of both AS and CS *gh2*, approximately 2000 bp upstream of the transcription initiation site (1897 bp in AS; 2057 bp in CS) (Figure 3). This transposon is not found in the promoters of *gh1 *for either species. This places the insertion of this transposon between the time of the gene duplication and when the two species diverged. A second Tc1-like transposon, DTSsa2 [Genbank:EF685955], is inserted approximately 7 kb upstream of the initiation site in only the AS *gh2 *promoter. In addition, a 1.2 kb fragment of SsaRT.3, a non-LTR long interspersed nuclear element (LINE) sequence identified in AS (unpublished data), is inserted approximately 1420 bp 5' of the transcription start site in CS *gh1 *(Figure 3). This fragment is truncated at the 3'-end, consistent with partial retrotranscription. Furthermore, an unusual insertion 10 kb upstream of only the CS *gh1 *gene was found (Figure 3). This insertion is approximately 20 kb in length containing a 10 kb palindromic sequence which is part of a Polinton-1 transposon (Figure 4) [21].

### Tissue expression PCRs

To examine the functionality of the genes identified on each *gh*-containing BAC, primers specific for each gene were designed and used in PCRs on a panel of tissues for each species [see Additional files 2 and 3]. This was not done for genes that were clearly pseudogenic or for genes for which information was incomplete, such as the genes that encode dynein heavy chain (data not shown) and microtubule associated protein Tau. Microtubule associated protein Tau is pseudogenic due to frameshifts and stop codons within the gene in each species (data not shown).

We conducted tissue expression studies of the 7 genes we had characterized in the BACs to determine if similarities existed in expression pattern between the two species; if expression was confined to specific tissues and whether indeed each of these genes were functional. Of the 7 genes examined, the expression of the *gh *paralogues was the least diverse. The expression of *gh1 *and *gh2 *was restricted to only muscle, brain, gill or eye (pituitary gland was not examined). Expression of the *scn *genes was not restricted to just skeletal muscle, but indeed was found in every tissue except the skin. It is also evident that *ifna1 *has fairly broad expression in the tissues examined here.

Myosin alkali light chain 1 also was expressed in a large range of different tissues. However, myosin alkali light chain 2 (*mlc2*) appears to not be expressed at detectable levels in any of the tissues we examined in either species, despite in silico analysis indicating the viability of the *mlc2 *transcript. Each gene that encodes *mlc2 *appears intact, with no obvious problems for transcription or translation.

## Discussion

### GH transcription

Transactivation and footprinting studies have been conducted on the *gh *promoters of both Chinook salmon and rainbow trout [22,23]. Assembly of at least part of the transcriptional machinery was delineated to binding elements between positions -300 to -30. It was demonstrated that cyclic-AMP/PKA regulation of these promoters required nucleation and recruitment of both pituitary transcriptional activator-1 (Pit-1) and cAMP-response element (CRE) binding proteins (CREB) [22,23].

We note strong sequence identity of potential Pit-1, CREB, and retinoic acid receptor (RAR)/retinoid × receptor (RXR) binding elements that form the core of the proximal *gh *promoters of both Atlantic salmon (AS) and Chinook salmon (CS). An asymmetric CRE (TGACG) at -217/-213 is flanked by at least four Pit-1 binding elements [see Additional file 1]. The more distal Pit-1 binding elements follow more strictly the core consensus sequence for Pit-1 (W_3_CAT) that is conserved in fish and mammals [24]. The potential RAR/RXR response element present at position -190/-171 could hypothetically permit interaction of chicken ovulbumin upstream promoter transcription factor (COUP-TF), or retinoic acid, estrogen or thyroid hormone receptors with an assembled Pit-1/CREB complex. Different variations of GTCT-rich blocks immediately upstream of each proximal promoter, as well as within the introns of each gene, may make regulatory or structural contributions to *gh *transcription. For example, some of the CTCT blocks found in these regions could serve as binding elements for GAGA-binding factors [25].

However, differences do exist between the AS and CS *gh1 *and *gh2 *promoters. For example, there is the potential for Pit-1 to bind a reverse-oriented response element roughly 250 bp further upstream in each *gh2 *promoter that is not present in the *gh1 *promoters. No palindromic CREs are found within 15 kb upstream of the *gh *genes, but one is present in intron 4 of the *gh1 *genes [see Additional file 1]. The different CRE, ERE and Pit-1 motif locations suggest that the *gh1 *and *gh2 *paralogues may have distinct regulatory modes and possibly act to function differently and/or at different times of salmonid development.

### GH expression and function

The more traditional models that have regarded divergence among duplicate genes as a means for acquisition of new functions have met with recent challenges. For example, it has been demonstrated that mutation in many duplicated genes of rice is much slower than in 'singletons' and less drastic in terms of amino acid change [26]. Conservation of the apparent crucial functionality of the duplicates outweighs processes that lead to neofunctionalizaton. Indeed, it may be more common for gene novelty to arise from preserved duplicates that are subfunctionalized [27].

At the amino acid level, CS GH1 appears to be undergoing change much faster than any of the other GHs (Figure 2). In terms of retention of amino acid residue identity, the CS GH1 ORF has more changes than any of the other translated regions (nine differences overall compared to no more than three for any other gene). These changes do alter the polarity and charge of the proteins and therefore may subtly effect the kinetics and affinity of hormone binding to their receptor(s). We did find colocalized expression of both cDNAs in the CS gill and brain [see Additional files 2 and 3]. Interestingly, both GH receptors have been found in various regions of the brain and in the gill filaments of trout [10]. Furthermore, although the very limited amount of change between all four genes indicates that their functions are conserved, we do observe that GH1 and GH2 may potentially have different subfunctions. Our RT-PCR work demonstrates that some tissue expression segregation has occurred, whereby only *gh2 *appears to be expressed in muscle or eye [see Additional files 2 and 3].

To determine the function of locally expressed *gh2 *in the gill, it will be necessary to elucidate the specific cells GH synthesis is associated with and to determine whether local production is a component of known osmoregulatory processes or some other activity (see [7]). It is also possible that the *gh2 *expression observed in the gill is from leukocyte accumulation as leukocytes have been shown to produce both *gh1 *and *gh2*, with *gh2 *the predominant transcript [28].

### Expression of genes linked with GH

Much less is known about the expression of the skeletal muscle sodium channel (*scn*), microtubule associated protein Tau (*mapt*), myosin alkali light chain (*mlc*) and interferon alpha-1 (*ifna1*) genes in salmonids. To the best of our knowledge, for the other genes examined in this study, only the promoters of two *ifn *genes have been characterized in salmon. Two different AS type 1 *ifn *genes have been demonstrated to each possess two promoters that respond to different phases of infection or are tissue-specific in their transcriptional contexts [29].

The *scn *gene has been characterized in zebrafish [30] and fugu [31]. Some subfunctional partitioning of the four sets of duplicated zebrafish *scn *genes does exist, but there is still expression overlap across neuronal and muscle tissues [32]. The expression of the *scn *genes in the salmonids examined here also are not restricted to skeletal muscle, but their activities are found in a large number of different tissues [see Additional files 2 and 3].

There appears to be a number of *ifn *genes in both the Atlantic and Chinook salmon GH1 BACs analyzed (data not shown). At least one functional *ifna1 *gene and two to five different *ifna *genes (that might be pseudo or partial genes) exist at these loci for each species. It should be noted, however, that this region was difficult to sequence and assemble due to repetitive regions, therefore we cannot conclude unequivocally on the number of *ifn *genes present at these loci. A genomic BAC clone containing the *ifna1 *gene and seven other *ifn *genes has been characterized in Atlantic salmon (Robertsen, B: 7^th ^International Symposium on Fish Immunology. Abstract, 2007). However, to date, we find no other *ifna *genes present in our database other than *ifna1 *to enable us to conclude that expression of these other *ifn *genes does occur. Zebrafish also has two to three *ifn *genes in the 3'-flanking region and the s*cn *gene in the 5'-flanking region of the *gh *gene on chromosome 3 [33].

### Genome rearrangements

The *gh2 *genes have undergone some rearrangements in comparison to the *gh1 *paralogues (differences in promoter sequences, partly due to variable transposon integrations; deletions or lack of microsatellite insertions in intron 3; intronic microsatellite variations in intron 4). Part of this may be due to chromosome structure or positioning [8]. Also, some of the genomic variations observed between the *gh1 *and *gh2 *paralogues might be due to vestigial effects caused by a rearrangement that led to the rise of the Y-linked *gh2 *pseudogene [34]. Whether the additional transposon insertions found in the AS *gh2 *promoter can account for why we do see *gh*-Y in CS, but not in AS [35], will require characterization of other Pacific salmon *gh *regions. It should be noted, as an example, that dramatic differences have also been found between the promoters of the salmon form of gonadotropin-releasing hormone in AS and sockeye salmon [36].

An indication of the changes that can occur when sequences of various transposon families integrate into the promoters of genes are shown in Figure 3. Insertions such as these may help to shape the dynamism of genomes by influencing modes of transcription through the introduction of new regulatory elements, by changing the distances between functional binding elements or by disabling them. It can be envisaged how alterations to transcription through these integration events could lead to changes in the function of their translated products over time. For example, note there are at least two different transposon sequences found within the AS *gh2 *proximal regulatory sequences that are not present in any of the other *gh *promoters (Figure 3). These integrated sequences might impact the observed specific expression of *gh2 *in AS compared to CS [see Additional files 2 and 3]. Also, the presence of a Tss transposon in the 5' sequence of each *gh2 *and an HpaI short interspersed nuclear element (SINE) in intron 3 of each *gh1 *is consistent with speciation events occurring after the gene or genome duplication events (Figures 3 and 4).

Vertebrate genomes contain large numbers of transposon sequences. The mouse and human genome contain approximately 3 million such sequences and zebrafish approximately 1.4 million. However, transposons occur in certain genome regions more frequently than in so-called transposon-free regions (which range from 5 to 66 kb in length), frequently associated with developmental genes [37,38]. This association with such genes suggests that transposons are deleterious to gene integrity possibly through increased mutagenesis or recombination, or by changing regulation. The presence of the Polinton sequence found upstream of the CS GH1 gene might therefore affect its genomic stability.

## Conclusion

This is one of the first in a series of studies that are needed to document coding and non-coding changes that have occurred subsequent to a whole genome duplication. As GH has been the focus of biotechnological advances in aquaculture, it is important to investigate expression changes and genomic organizational changes in important economic traits such as growth. In this paper, we report on the impact of repeat elements and transposon integrations and show that in *Oncorhynchus *species the GH1 duplicate has undergone a higher rate of change.

Genomes, particularly salmonid genomes, are dynamic. Part of the dynamism is the result of the purported whole genome duplication and the past integration of SINEs and LINEs into them. LINEs and SINEs introduce repetitive elements that may misalign causing unequal recombination to occur, and thereby introduce deletions and insertions among duplicated genomes. Coupled with this is the integration of a wide assortment of transposon sequences as described here and elsewhere [19]. Over time, these genomic rearrangements have led to the creation of pseudogenes, structural differences between duplicated genes and to differential regulation of paralogues.

In AS and CS, we show that transposon insertions have occurred differently in the promoters of *gh*, within and between each species. The organization of transposon families in the BACs containing *gh1 *and in the BACs containing *gh2*, is very similar within orthologous regions (Figure 4). However, much less conservation is apparent in comparisons between the *gh1*- and *gh2*-containing paralogous BACs for the two fish species (Figure 4). The appearance of repeated elements and differential rates of change in the *gh1 *and *gh2 *regions is consistent with the hypothesis that a burst of transposition activity occurred during the speciation events which led to Atlantic and Pacific salmon [19]. Genome and gene duplication (30 to 100 MYA; [39,40]) has taken place much earlier than the speciation (14 to 23 MYA; [9,41,42]) and these data suggest that after gene duplication, *gh1 *in *Oncorhynchus *has evolved much faster than *gh2*, possibly because the genomic region for *gh1 *has undergone more reorganization compared to the region containing *gh2*.

## Methods

Atlantic salmon (AS) CHORI-214 [43] and Chinook salmon (CS) CHORI-217 [44] bacterial artificial chromosome (BAC) libraries were obtained from BACPAC Resources, Children's Hospital Oakland Research Institute (CHORI) [45]. AS BAC library filters were hybridized with an oligonucleotide probe (5'-TCCCAAACAAACAGCAACATACTCAACCGACCACCGCACT-3') designed from the AS *gh1 *EST [GenBank:X61938] that had been end-labeled with gamma-^32^P-ATP (Amersham). The CS BAC library filters were screened using a *gh *cDNA probe (GH2-8) generated from sockeye salmon genomic sequence by a PCR-based intron deletion methodology [35]. The CS GH2-8 probe was labelled with alpha-^32^P dCTP using the RediprimeII Random Prime Labelling system, and purified through a Probequant G-50 micro column (Amersham). Filter hybridizations were conducted as described by CHORI [45]. Probed BAC library filters were visualized using a Molecular Dynamics Storm PhosphorImaging system.

### BAC DNA confirmation

Confirmation of AS *gh*-containing BACs was performed by comparisons of *Hind*III restriction digests of the isolated clones to *in silico *digests for each BAC. The entire *Salmo salar *genome BAC library has been digested by *Hind*III and fragment profiles are available on the internet Contig Explorer version 3.4 (iCE 3.4) database [14]. Fingerprinting of the CS BACs was carried out by SnaPshot (ABI) labeling of restriction-digested fragments and samples were analyzed on an ABI 3130xl genetic analyzer. Data was processed utilizing Genemapper and fingerprinted contigs assembled as described by Luo et al [46].

BACs were assessed for whether they contained *gh1 *or *gh2 *using gene-specific polymerase chain reactions (PCRs). Primer sets were designed for AS *gh1 *intron 2 (5'-AAAACCAACGGCTCTTCAAC-3' and 5'-GGAGTCAGAGTTACAGAAGTCCAG-3'), intron 5 (5'-GATGACAATGACTCTCAGCAGC-3' and 5'-TGTATCTGGGAAACCGAACC-3'), as well as for *gh2 *intron 3 (5'-ATCGTGAGCCCAATCGACAAGCAG-3' and 5'-GGGTACTCCCAGGATTCAATCAGG-3'). Primer sets were also designed for exon 3 (5'-ACATGCAGCAGGATGCTAAG-3' and 5'-TTTCAGACCTTTATTGTCATCACC-3') and exon 5 (5'-GGTTCTGTGGACACTCAGTCC-3' and 5'-TCTTCGGAGGTGGCAAAG-3') of the sodium channel (*scn*) upstream of the *gh *genes. Detection of the *gh *genes in the CS BACs was confirmed using the primer set 5'-AGCCTGGATGACAATGACTC-3' and 5'-CTACAGAGTGCAGTTGGACT-3' which can distinguish all forms of *gh *in the genome (GH1, GH2, and GH-P; [35]) and for the *scn *gene with 5'-TTCCGCCACTTCACCCTTG-3' and 5'-AGGGGCGTGTTGAACAGCTC-3'.

PCRs were performed using 200 ng of BAC DNA utilizing hot start PCRs: reaction mixtures without any enzyme were heated to 94°C for 3 min, cooled to 80°C and then 0.625 U of *Taq *DNA polymerase (Invitrogen) was added. Each PCR then underwent 35 cycles of the following parameters: 94°C for 20 sec, 50°C for 30 sec and 72°C for 45 sec. AS *gh1 *was contained on BACs 11-I-04 and 73-D-15 and *gh2 *in BAC 63-I-10. The CS *gh1 *was localized to BAC 108-O-24 and *gh2 *to BAC 206-E-17.

### BAC preparation and library construction

BAC DNA was isolated by an alkaline lysis procedure using Nucleobond columns (Clontech) following the manufacturer's protocol. The isolated BAC DNA was nebulized and the DNA ends were made blunt by filling with T4 polymerase. The blunt-ended, repaired DNA was size fractioned by electrophoresis and the gel region corresponding to 1.6 to 4.0 kb was excised and gel purified (Qiagen). The fragments were blunt-end ligated into pUC19 plasmid cut with *Hinc*II (NEB) and transformed into electrocompetent DH5α *E. coli *cells using a Bio-Rad Gene Pulser system. Library quality was evaluated and high redundancy plating was followed by large-scale colony picking (Genetix). Extracted recombinant plasmid templates were sequenced on an ABI 3730 DNA sequencer.

### BAC contig assembly

Bases were called using PHRED [47,48]. High quality sequence reads were assembled using PHRAP [49] and then viewed and edited using Consed [50]. Some gaps in BAC assembly were filled by designing primers to the contiguous sequence ends, followed by amplification of the BAC region by PCR and subsequent cloning and sequencing of the fragments. Each BAC has been deposited in GenBank as follows: AS GH1 BAC 11-I-04 and BAC 73-D-15 combined [GenBank:EU621898]; AS GH2 BAC 63-I-10 [GenBank:EU621899]; CS GH1 BAC 108-O-24 [GenBank:EU621900] and CS GH2 BAC 206-E-17 [GenBank:EU621901].

Dotter [51] and PipMaker [52] were used to compare each BAC sequence to itself and to identify duplicated and repeated regions. Identification of other repeat elements was done with RepeatMasker [18] using repeat library 4.01 from Repbase [53], as well as a salmonid repeat database [19]. Gene location and direction on each BAC was determined using Digit Integrated Gene Identification Tools (DIGIT) [15].

### GH alignment, annotation, and comparison

AS *gh1 *[GenBank:AY614010] and *gh2 *[GenBank:M21573] gene sequences were used to align the *gh1 *and *gh2 *paralogues with BioEdit [54]. BioEdit was also used to annotate the two paralogues and calculate percent similarities between the two genes, their coding sequences, and their amino acid sequences. Searches and comparisons of regulatory elements for the two paralogues were also performed using BioEdit. Identification of transposon insertions was performed using Dotter plots, comparing 15 kb of each promoter region with salmon transposon sequences [19].

### Reverse transcription and cDNA amplification

Total RNA was extracted in TRIzol reagent (Invitrogen) from flash-frozen, adult AS (Mowi stock, DFO, West Vancouver) and CS (Chehalis River Hatchery) kidney, muscle, skin, gut, gill, spleen, brain, heart, testis, liver, eye and pyloric caecum tissues. The extracted total RNAs were cleaned using MEGAclear (Ambion) and then quantified and quality-checked by spectrophotometer and agarose gel, respectively.

The cDNAs were synthesized in 25-μL reactions from 1.0 μg total RNA using oligo(dT)_15 _(Promega) and Supercript II RNase H^- ^reverse transcriptase according to the manufacturer's instructions (Invitrogen). The reactions were incubated at 37°C for 90 min and the transcriptase heat-inactivated at 70°C for 30 min. Approximately 200 ng of cDNA was used in each 25-μL PCR reaction containing 1.25 U Taq polymerase, 1 × Taq buffer, 1.25 mM MgCl_2_, 10 mM dNTPs (Invitrogen) and 15 pmol of each gene-specific 5' and 3' primer [see Additional file 4]. Each PCR was carried out under the following cycling parameters: 94°C for 2 min, then 35 cycles of 94°C for 30 sec, 55°C for 30 sec, and 72°C for 1 min using a Perkin Elmer 9600. The AS *scn1 *and *scn2 *PCRs were similarly amplified but the anneal temperature used was 57.5°C. The integrity of each cDNA used was confirmed by control PCRs using ubiquitin primers.

The PCR products were separated by electrophoresis on 1.25 to 1.30% agarose gels and photographs were stored using an UVP GelDoc-It documentation system (UVP). Representative products were isolated and cloned into pCR2.1-TOPO vector (Invitrogen) and sequenced to confirm gene identities.

## Authors' contributions

KRVS: performed tissue expression RT-PCRs, *gh *promoter analysis and drafted the manuscript. RY: performed BAC sequence data analysis and annotations. JdB: performed transposable element analysis. KPL: designed probes and performed hybridizations to identify AS *gh*-containing BACs. BG: designed probes and performed hybridizations to identify CS *gh*-containing BACs. CAS: performed initial sequencing and analysis of Atlantic salmon *gh*-containing BACs for Biology Honours thesis MRB-S: and AR: performed clone preparation and sequencing. WSD, RHD and BFK: obtained funding, and contributed to experimental design, analysis and writing of the manuscript. All authors read and approved the final manuscript.

## Supplementary Material

Additional file 1 **Comparison of Atlantic and Chinook salmon growth hormone 1 and 2 genes.** Exons are shaded in red. Potential transcription factor binding sites and poly(A) termination signals are boxed. Characteristic insertions or deletions reported by McKay et al. (2004) are underlined. Click here for file

Additional file 2 **Reverse transcriptase PCR validation and cDNA expression profiles in twelve different tissues.** Reverse transcriptase PCR validation and cDNA expression profiles in twelve different tissues: 1: kidney, 2: muscle, 3: skin, 4: gut, 5: gill, 6: spleen, 7: brain, 8: heart, 9: testis, 10: liver, 11: eye and 12: pyloric caecum. The integrity of each cDNA used was confirmed by control PCRs using ubiquitin primer set. For each gene-specific PCR experiment, a negative control with no template (NC) was included. Abbreviations for gene names are as follows: GH: growth hormone; Scn: skeletal muscle sodium channel alpha subunit; Mlc: myosin alkali light chain; IFN: interferon. The strongest marker band indicates a fragment length of 500 bp. Click here for file

Additional file 3 **Reverse transcriptase PCR validation and cDNA expression profiles in twelve different tissues.** Reverse transcriptase PCR validation and cDNA expression profiles in twelve different tissues: 1: kidney, 2: muscle, 3: skin, 4: gut, 5: gill, 6: spleen, 7: brain, 8: heart, 9: testis, 10: liver, 11: eye and 12: pyloric caecum. The integrity of each cDNA used was confirmed by control PCRs using ubiquitin primer set. For each gene-specific PCR experiment, a negative control with no template (NC) was included. Abbreviations for gene names are as follows: GH: growth hormone; Scn: skeletal muscle sodium channel alpha subunit; Mlc: myosin alkali light chain; IFN: interferon. The strongest marker band indicates a fragment length of 500 bp. Click here for file

Additional file 4 **Gene-specific primer oligonucleotide sequences used in tissue expression studies.** A list of primer identification and primer sequences for each amplified gene of interest are provided. Click here for file
