# Supplementary material for: Isolation, characterization and comparison of Atlantic and Chinook salmon growth hormone 1 and 2
Source: BMC Genomics. 2008 Nov 3;9:522. doi: 10.1186/1471-2164-9-522 (PMC2584663; doi:10.1186/1471-2164-9-522)
Supplement: Additional file 1 — Comparison of Atlantic and Chinook salmon growth hormone 1 and 2 genes. Exons are shaded in red. Potential transcription factor binding sites and poly(A) termination signals are boxed. Characteristic insertions or deletions reported by McKay et al. (2004) are underlined. [file 1471-2164-9-522-S1.doc]

. . . .10 . . . .20 . . . .30 . . . .40 . . . .50 . . . .60

ASGH1 1:.....GTGCCTAATCTG.CAGTAATACATGCAGGCCTTTCTCTTGCATTTCAA.AGATGA: 53

CSGH1 1:.....TCAATCAATCAATCAACGGATGAAGTAGGGCCTGCACACTTGCTCCAATAGATAC: 55

CSGH2 1:AAAAAATCATTAAAGGCACATCGATTTTTTTTACCCCAAAATCAATCAATCAATCAATGA: 60

ASGH2 1:........CTTAACGGCATATAGGTTT...TTACCCCAAAATCAATCAATCAATCAATGA: 49

. . . .70 . . . .80 . . . .90 . . . 100 . . . 110 . . . 120

ASGH1 54:GAA.....GAAAAAAAAAATATAAACCTGCATGTTTTTTTCTTTGTATTATCGTCTACCA: 108

CSGH1 56:CGT.....TATTAGGCTTTCTTGACAATGTTGAGATTACTGAAAGGATCTTCGTCAGGTA: 110

CSGH2 61:AGTA....GGGCCTACACACTTGCTCCAACAGATCGCTTTTATTGGG.CTTCGTCGGGTA: 115

ASGH2 50:AGTAGGTAGGGCCTACACACTTGCTCCAACAGATACCTTTATTGGG..CTTCGTCAGGTA: 107

. . . 130 . . . 140 . . . 150 . . . 160 . . . 170 . . . 180

ASGH1 109:GATCTAATG.TGTTATATTCGCCTACATTACTTTCACATTTCCACAAACT........CC: 159

CSGH1 111:TGACAAAGGCTCTCACACAAAAAGAAAAAGGATCGGGTATTTCACAGACTGGTATTGATC: 170

CSGH2 116:TAACAAAGGCTCTCACACAAAAAGACAAAG...CGGGTATT...............GATC: 157

ASGH2 108:TAACAAAGGCTCTCGCACAAAAATAAAAAG...TGGGTATTTCACGGACTGGTATTGATC: 164

. . . 190 . . . 200 . . . 210 . . . 220 . . . 230 . . . 240

ASGH1 160:AAAGTATTTCCTTTCAAATG.GTATCAAGAATATGCATATCCTTGCTTCAGGTCCTGAGC: 218

CSGH1 171:AAGTGACTCTTTATGTTGTGTGTGTTGATGATAACAAAGACCCTGTCT..GAATTTAAAC: 228

CSGH2 158:AAGTGGCTGTTTATGTTATGTAAACGAACAAAAGACATA.CTTTGT..............: 202

ASGH2 165:AAGTGACTGTTTATGTTATTTAAACAAATAGAAAACATA.CCTTCT..............: 209

**Pit-1**

. . . 250 . . . 260 . . . 270 . . . 280 . . . 290 . . . 300

ASGH1 219:TACAGGCAGTTAGATTTGGT.TATGTCATTTCAGGCGAAAATTGGGAAAAAAAGGGTCCG: 277

CSGH1 229:AAAAAACGATACATTCTAACATGTGCCGTCTCGAGTCCTTCTCTGTGTGTCTACTTTGAG: 288

CSGH2 202:...AAGATGTGCGTCTCAAG....TCCTTGATAGGTCCTTCTCTGTGTGTCTACCTTGAG: 255

ASGH2 209:.................AAGATGTGCC.TCTCAAGTCCTTCTCTGTGTGTCTACTTTGAG: 251

. . . 310 . . . 320 . . . 330 . . . 340 . . . 350 . . . 360

ASGH1 278:ATCCTTAAG.AGGTTTTAATGCCATAGGACATTCAATTTGACAATAAACAATAAAATATT: 336

CSGH1 289:GAATTTGACTAAGTGTTAATGCCATAGGACATTCAATTTGACATTAAACAATAACATATT: 348

CSGH2 256:GTCT........GTGCTAATACCATAGGACATTCAATT.GACATTAAACAATCAAATATT: 306

ASGH2 252:GTCT........GTGCTAACACCATAGGACATTCAATT.GACATTAAACAATAAAATATT: 302

. . . 370 . . . 380 . . . 390 . . . 400 . . . 410 . . . 420

ASGH1 337:GGTGCTGATAAAGAAGCAATATAATACATTTGTCAA.ATACTGCATGTTATCTACAGTAC: 395

CSGH1 349:GGGGTTAATAAAGAAGCAATATAATAAATGTCTTGTCATACTGCCTGTTATCTACAGTAC: 408

CSGH2 307:GTTGCTAATAAAGAAGCAATATAATATATTTGTCAA.ATAATGCCTGTTATCTACAGTAC: 365

ASGH2 303:GGTGCTAATAAAGAAGCAATATAATACATTTGTCAA.ATAATGGCTGTTATCTACAGTAC: 361

. . . 430 . . . 440 . . . 450 . . . 460 . . . 470 . . . 480

ASGH1 396:CACAGGTGGAATGGCAGAATAACCGGTGT...............................: 424

CSGH1 409:CACAG.CAGAATGGCAGAATAACCTGTGTGTGTGTGTGTGTGTGTGTGTGTGTGTGTGTG: 467

CSGH2 366:CACAG.CGGGATGGCAGAATAACCGGCGTTGTTGTCAAGTTACAGGGGT...........: 413

ASGH2 362:CACAG.CGGAATGGCAGAATGACCGGTGTTGTCGTCAAGTTACGG...............: 405

. . . 490 . . . 500 . . . 510 . . . 520 . . . 530 . . . 540

ASGH1 424:....................TGTGTGTGTAACTTGTGTCCATTCATTACATCCTAGACAA: 464

CSGH1 468:TGTGTGTGTGTGTGTGTGTGTGTGTGTGTATCTTATGTCCATTCATTACATCCTAGACAA: 527

CSGH2 413:....TGTGTCTCTCTGTGTGACTGAGTGTAACTTTTGTTCATTCATTATGTCCTAGACAA: 469

ASGH2 405:..TGTGTGTGTCTGTCTGTGTGTGAGTGTCACTTTTGTCCATTCATTACATCCTAGACAA: 463

**Pit-1**

. . . 550 . . . 560 . . . 570 . . . 580 . . . 590 . . . 600

ASGH1 465:CAGAGGTTTGTGTTGT....GTTTTGACCCTAATTCGTTCAGTCATCAAGTAAGTTGTT.: 519

CSGH1 528:CAGAGGTTTGTGTTGTATGTGTTTTGACCCTAATTCGTTCAGTCATCAAGTAAGTTGTT.: 586

CSGH2 470:CAGAGGTTTGTGACGTATGTGTTTTGACCCTCATTTGTCATGTCATCGGGTACGTTTTTG: 529

ASGH2 464:CAGAGGTTTGTGTTGTATGTGTTTTGACCCTAATTTTTCAAGTCATCGAGTACGTTTTT.: 522

**½** **RARE**

. . . 610 . . . 620 . . . 630 . . . 640 . . . 650 . . . 660

ASGH1 520:TTTT.AGGACACGTCCCCTCTTCC.AAACTCATGGAAAAATGTAGGATTGATTTGACGCA: 577

CSGH1 587:TTTTTAGGACACCTCCCCTCTTCCCAAACTCATGGAAAAATGTATGATTGATTTGACGTA: 646

CSGH2 530:TTTTTAGGA...GTCACCTCTTCCCGAACTCATGGAAAAATGAATGATTGATTTGACACA: 586

ASGH2 522:..TTTAGGACAGGTCACCTCTTCCCGAACTCATGGAAAAATTCATGATTGATTTGACGCA: 580

**Pit-1 CRE**

. . . 670 . . . 680 . . . 690 . . . 700 . . . 710 . . . 720

ASGH1 578:TTATAGTGATTGTTCCACAATCACATACAAAAACAGGTCCTATTAATGAAAGGTGGTAAA: 637

CSGH1 647:ATATGGTAATTGTTCCGTCATCACATACAAAAACAGGTCCTATCAATGAAAGGTGGTAAA: 706

CSGH2 587:TCATACTGATTGTTCCATCGTCACATACAAAAACCGGTCCCATCGGCGAGAGGTGGTACA: 646

ASGH2 581:TTACACTGATTGTTCCACAATCACATAGAAAAACAGGTCCCTTCAACGAAAGGTGGTAAA: 640

**RAR/RXR binding element**

. . . 730 . . . 740 . . . 750 . . . 760 . . . 770 . . . 780

ASGH1 638:TGGATGAAAATCTCATGTTTCCTCCTGGTGATACATTAAAACATGGGTTCCCCATCTATA: 697

CSGH1 707:TGGATGAAAATGTCATGTTTCCTCCCATTGATACATTAAAACATGGATTCCCCATCTATA: 766

CSGH2 647:TGGA.GAAAATCTCATGTTTCCTCCTGTTGATACATTAAAACATGTGTTCTCCATCTATA: 705

ASGH2 641:TGGA.GGAAATCTCATGTTTCCTCCTGTTGATACATTAAAACATGGGTTATCCATCTATA: 699

**Pit-1 Pit-1 TATA box**

. . . 790 . . . 800 . . . 810 . . . 820 . . . 830 . . . 840

ASGH1 698:AAAACAGTGGTCCCAAACAAACAGCAACATACTCAACCGACCACCGCACTTTCAAGTTAA: 757

CSGH1 767:AAAACAGTGGCCCCAAACAAACGACAACATACTCAACCGACCACCGCACTTTCAAGTTAA: 826

CSGH2 706:AAAACAGGGGCCCCAAACAAGCGGCAACATACTGAACCGACCACCACACTTTCAAGTGAA: 765

ASGH2 700:AAAACAGTGGCCCCAAACAAACGGCAACATACTCAACCGACCACCGCACTTTCACGTGAA: 759

**TATA box**

. . . 850 . . . 860 . . . 870 . . . 880 . . . 890 . . . 900

ASGH1 758:GTAATCATCCTTGGCAATTAAGAGTAAAA.**ATG**GGACAAGGTAAGCCTGCTTTTTCTGTC: 816

CSGH1 827:GTAACCATCCTTGGCAATTAAGAGTAAAA.**ATG**GGACAAGGTAAGCCTGCTTTTTCTGTC: 885

CSGH2 766:GTAATCATCCTTGGCAATTAAGAGAAGAA.**ATG**GGACAAGGTAAACCAGCTTTTATT...: 821

ASGH2 760:GTAATCATCCTTGGCAATTAAGAGAAAAAA**ATG**GGACAAGGTAAACCAGCTTTTATT...: 816

Exon 1

. . . 910 . . . 920 . . . 930 . . . 940 . . . 950 . . . 960

ASGH1 817:TATTTCTTTTTTCAGTGGGAAGTCAGTGTACCATTTAGTACAATTTAACTTACACATTTA: 876

CSGH1 886:TATTTCTTTTTTCAGTGGGAAGTCAGAGTACCATTTAGTACAATTTAACT..........: 935

CSGH2 821:..TTATTTTTTTAAGTGGGAAGTCAGTGTACCATTTAATACCATTTAACTTTAACATTTA: 879

ASGH2 816:...TTCTTTTTTAAGTG..AAGTCAGTGTACCATTTAATACCATTTAACTTAAACATTTA: 871

. . . 970 . . . 980 . . . 990 . . .1000 . . .1010 . . .1020

ASGH1 877:ATCACTGAGGCAGGGGCCAACACGGCAGAGAAA.AGTGAACAAGTATTCTACTACTATGA: 935

CSGH1 935:..................................................ACTGCTATGA: 945

CSGH2 880:GTTACTGAGGCAGGGGCCAAGAAGGCAGAGAAAGAGTGAACAAGTAATGTACTGCCATGA: 939

ASGH2 872:ATCCCTGAGGCAGGGGCCAACAAGGCAGAGAAAGAGTGAACAAGTAATTTACTGCCATGA: 931

. . .1030 . . .1040 . . .1050 . . .1060 . . .1070 . . .1080

ASGH1 936:GGTTATAAATCTATTGACACAGAACCACCTGCTTTAACAACCTAACTATGTGATCTATAA: 995

CSGH1 946:GGTTATAA.TCTATTGACACAGAACCACCTGCTTTAACAACCTAACTATGTGATCCATAA:1004

CSGH2 940:GGGTATAA.TCTACTTACACAGAACCACTTCCTTTAACAACCTAACCAGATGATCTATTA: 998

ASGH2 932:GGGTATAA.TCTACTGACACATAACCACTTCCTTTAACAACCTAGCTATGTGATCTATTA: 990

. . .1090 . . .1100 . . .1110 . . .1120 . . .1130 . . .1140

ASGH1 996:CATTTACATTTGAGTCGTTTAGCAGACGCTCTTATCCAGAGCGACTTACAGGAGCAATTA:1055

CSGH1 1005:CATTTACATTTTTGTCATTTAGCAGACACTCTTTTCCAGAGCGACTTACATGAGCAATTG:1064

CSGH2 999:GTTTTACATTTGAGTTATTTAGCAGAGACTCTTATCCAGAGCGACTTCCAGGAGCAATTA:1058

ASGH2 991:GATTTACATTTGAGTTATTTAGCAGACACTCTTATCCAGAGCGACTTACAGGAGCAATTA:1050

. . .1150 . . .1160 . . .1170 . . .1180 . . .1190 . . .1200

ASGH1 1056:GGGTTAAGTGCCTTGCTCAAGGGCACGTCGACAGATTTCTCACCTAGTCAGCTCAGGGAT:1115

CSGH1 1065:GGGTTACGTGCCTTGCTCAAGGGCACATC...AGATTTCTCACCTAGTCAGCTCTGGGGT:1121

CSGH2 1059:GGGTTAAGTGCCTAGCTCAAGGGCACATCAACAGATTTTTCACCTAGTCAGCTCAGCTCT:1118

ASGH2 1051:GGGTTAAGTGCCTTGCTCAAGGGCACATCAACAGATTTATCACCTAGTCAGCTCAGGGAT:1110

. . .1210 . . .1220 . . .1230 . . .1240 . . .1250 . . .1260

ASGH1 1116:TGAA.ACCAGTAACCTTTCAATTACTTACCCAACGCTCTTAACC.GCTAGGCTATTGGTG:1173

CSGH1 1122:TGAA.ACCAGTAACCTTTCAGTTACTGACCCAGCGCTCTTAACCAGCTAGGCTATTGGTG:1180

CSGH2 1119:CCAACGCTCTTAATCGCTAGGCTATTGAGAAAGA.....TAGCAAATTGAG.....AATA:1168

ASGH2 1111:TCAA.ACCAGTAACCTTTCAGTTACTGGCTCAACGCTCTTAATC.GCTAGGCTATTGGTG:1168

. . .1270 . . .1280 . . .1290 . . .1300 . . .1310 . . .1320

ASGH1 1174:TTCGATGGCTGAGAATATCTAACTAATGTATCTCACCATAATTCGACTTACTCGTTTT.A:1232

CSGH1 1181:TACGATGGCTGGGAAAATCTTACTAAGGTATCTCACCATAATTCGACTTACTCGTTTT.C:1239

CSGH2 1169:TCTTACTATTGAGAATATCTTACTAACATGTCGCAACATAATTTGACTTACTCGTTTTTA:1228

ASGH2 1169:TTCATAGGCTGAGAATATCTTACTAACATGTCGCAACATAATTTGACTTACTCGTATT.A:1227

. . .1330 . . .1340 . . .1350 . . .1360 . . .1370 . . .1380

ASGH1 1233:TACATTTGTTATTTTC.....TCTTTCTTTTAGTGTTTCTGCTGATGCCAGTCTTACTGG:1287

CSGH1 1240:TACATTTGTTATTTGAA....TCTCTCTTTTAGTGTTTCTGCTGATGCCAGTCTTACTGG:1295

CSGH2 1229:TACATTTCTTATTTTCTTTCATCTCTCTTTTAGTGTTTCTGCTGATGCCAGTCTTACTGG:1288

ASGH2 1228:TACATTTCTTATTTTCTTTCATCTCTCTTTTAGTGTTTCTGCTGATGCCAGTCTTACTGG:1287

Exon 2

. . .1390 . . .1400 . . .1410 . . .1420 . . .1430 . . .1440

ASGH1 1288:TCAGTTGTTTTCTGAGCCAAGGGGCAGCGATGGAAAACCAACGGCTCTTCAACATCGCGG:1347

CSGH1 1296:TCAGTTGTTTCCTGAGTCAAGGGGCAGCGATAGAAAACCAACGGCTCTTCAACATCGCGG:1355

CSGH2 1289:TCAGTTGTTTCCTGAGTCAAGGGGCGGCGATGGAAAACCAACGGCTCTTCAACATCGCGG:1348

ASGH2 1288:TCAGTTGTTTTCTGAGTCAAGGGGCAGCGATGGAAAACCAACGGCTCTTCAACATCGCGG:1347

. . .1450 . . .1460 . . .1470 . . .1480 . . .1490 . . .1500

ASGH1 1348:TCAACCGGGTGCAACATCTCCACCTAATGGCTCAGAAGATGTTCAATGACTTTGTAAGAC:1407

CSGH1 1356:TCAGCCGGGTGCAACATCTCCACCTATTGGCTCAGAAAATGTTCAATGACTTTGTAAGAC:1415

CSGH2 1349:TCAACCGGGTGCAACACCTCCACCTTTTGGCTCAGAAAATGTTCAACGACTTTGTAAGAC:1408

ASGH2 1348:TCAACCGGGTGCAACATCTCCACCTACTGGCTCAGAAAATGTTCAATGACTTTGTAAGAC:1407

. . .1510 . . .1520 . . .1530 . . .1540 . . .1550 . . .1560

ASGH1 1408:AGCTTTTGAATCTTCTTTTGACATATCAAATAGTGTATCAATGATTGTTCTTCTTCTT.G:1466

CSGH1 1416:AGCTTTTGAATCTTCTTTGGACATATCAAATAGTGTATCAATGATTGTTCTTCTTCTT.G:1474

CSGH2 1409:AGCTTTTGAATCTTCTTTTGACACAGCGGATAATGTTTCAGAGGTGGTTCCTCTTCTTTG:1468

ASGH2 1408:AGCTTTTGAATCTTCTTTTGACATAGCAAATAACGTTTCAAAGATTCTTCTTCTTCTT.G:1466

. . .1570 . . .1580 . . .1590 . . .1600 . . .1610 . . .1620

ASGH1 1467:TAGACA.GTGTCCTCTTTACACAACCCTCGTGGCAACAACAAAAAAATCTCTCTCCCTTC:1525

CSGH1 1475:TAGACA.GTGTCCTCTTCACACAACCCTCGTGGC.....TAAAAGAATCTCTCTCTCC.C:1527

CSGH2 1469:TAGACAAGTGTCCTCTTCACGCAAACCGAGCGGC......AAAACATTCTCTCTCCTGTC:1522

ASGH2 1467:TAGA...GTGTCCTCTTCACACAAACCTAGCGGCA.....AAAAAATTCTCTCTCCCGTC:1518

. . .1630 . . .1640 . . .1650 . . .1660 . . .1670 . . .1680

ASGH1 1526:TTTGTGATTTTGTGCAGGAAGGTACCCTGTTGCCTGATGAACGCAGACAGCTGAACAAGA:1585

CSGH1 1528:TTTGTGATTTTGTGCAGGACGGTACCCTGTTGCCTGATGAACGCAGACAGCTGAACAAGA:1587

CSGH2 1523:TTTGTGATTTTGTGCAGGAAGGCACCCTGTTGTCTGATGAACGCAGACAGCTGAACAAGA:1582

ASGH2 1519:TTTGTGATTTTGTGCAGGAAGGCACCCTGTTGTCTGATGAACGCAGACAGCTGAACAAGA:1578

Exon 3

. . .1690 . . .1700 . . .1710 . . .1720 . . .1730 . . .1740

ASGH1 1586:TATTCCTGCTGGACTTCTGTAACTCTGACTCCATCGTGAGCCCAATCGACAAGCTTGAGA:1645

CSGH1 1588:TATTCCTGCTGGACTTCTGTAACTCTGACTCCATCGTGAGCCCAGTCGACAAGCACGAGA:1647

CSGH2 1583:TATTCCTGGTGGACTTCTGTAACTCTGACTCCATCGTGAGCCCAATCGACAAGCAGGAGA:1642

ASGH2 1579:TATTCCTGCTGGACTTCTGTAACTCGGACTCCATCGTGAGCCCAATCGACAAGCAGGAGA:1638

. . .1750 . . .1760 . . .1770 . . .1780 . . .1790 . . .1800

ASGH1 1646:CTCAGAAGAGTTCAGTAAGTAACCTGGTTGAGACAATTATGCATGTTATGCCCTTTAAAA:1705

CSGH1 1648:CTCAGAAGAGTTCAGTAAGTAACCTGGCTGAGACAATTACGCATGTTATGCCCTTTAGAA:1707

CSGH2 1643:CTCAGAAGAGTTCAGTAAGTTACCTGGCTGAGACAAT.......................:1679

ASGH2 1639:CTCAGAAGAGTTCAGTAAGTTACCTGGCTGAGACAAT.......................:1675

. . .1810 . . .1820 . . .1830 . . .1840 . . .1850 . . .1860

ASGH1 1706:CCATATAAAAGTGTCAAATTGTGACAGGTCCACTCTGCTATTCACCTTAAATATGAATTC:1765

CSGH1 1708:CCATATAAA.GTGTCAAATCGTGACAGTTCCACTCTGCTATTCACCTTAAATATGAACTC:1766

CSGH2 1679:...........................................................C:1680

ASGH2 1675:...........................................................C:1676

. . .1870 . . .1880 . . .1890 . . .1900 . . .1910 . . .1920

ASGH1 1766:CTCCATGATGCATGATTACAAAA.TAAATAATATGGCATCTCAATTTGAACAATCGATAG:1824

CSGH1 1767:CTCCATGATGCAAGATTCCAAAAATAAATAATAGGGCATCTCAATTTGAACAATCGATAG:1826

CSGH2 1681:CTCCATGATGCACAATTCCAACA.TGAATAATAGGGCATCTCAATTTGAACAA.......:1732

ASGH2 1677:CTCCATGATGCATGATTCCAAAA.TAAATAATAGGGCATCTCAATTTGAACAATCGATAC:1735

. . .1930 . . .1940 . . .1950 . . .1960 . . .1970 . . .1980

ASGH1 1825:AACTTAGTCATTAGTTATTGGGAAAGCAGACCACCAATTATCTAAACTCCAATTTATAAA:1884

CSGH1 1827:AACTTAGTCATTAGTTATTGGGCAAGCAGACCACCAATTATGTAAACTCAAATTTATAAT:1886

CSGH2 1732:......GTCATTAGTTATTGGGCAAGCAGATCCCCGATTGTCTAAACTCCA.........:1777

ASGH2 1736:AACTTAGTCATTAGTTATTGGGCAAGCAGATCCCCGATTGTGTAAACTCCA.........:1786

. . .1990 . . .2000 . . .2010 . . .2020 . . .2030 . . .2040

ASGH1 1885:TGTTTTAATTTGAATTTTTTT...ACCATTATTTAACTAGGCAAGTCAATTAAGAACAAA:1941

CSGH1 1886:..TTTTTATTTAAATTTTATTTGAGCCTTTAATTAACTTGGCAAGTCAGTTAAGAACAAA:1944

CSGH2 1777:.....................................TGGGTAAAT.........ATATA:1791

ASGH2 1786:.....................................TGGGTAAAT.........ATATA:1800

. . .2050 . . .2060 . . .2070 . . .2080 . . .2090 . . .2100

ASGH1 1942:TTCTCATTTACAATGACAAGCAGAGGCTGCATCATGCATGGCTGTCGAGTGGCGCAGCAG:2001

CSGH1 1945:TTCTCATTTACAATGACAAGCAGAGGCAGCATCATGCATGGCTCTCGAGTGGCACAGCAG:2004

CSGH2 1792:CTGTA.........GATAAGCAGAACCAGCATCTTGCATGGT..................:1824

ASGH2 1801:CTGTA.........GATTAGCAGAGCCAGCATCATGCATGGT..................:1833

. . .2110 . . .2120 . . .2130 . . .2140 . . .2150 . . .2160

ASGH1 2002:TCTAAGGCACTGCATCTCAGTGTTAGAGGTGTCACTACAGACCCTGGTTCGATTCCAGAC:2061

CSGH1 2005:TCTAAGGCACTACATCTCAGTGCCAGAGGTGTCACTGCAGACCCTAGTTCGATTCCAGAC:2064

CSGH2 1824:....GGAAATTAAATCTA............GCCATGACAGGA........AGTTTTAAAT:1860

ASGH2 1833:....GGAAATTAAATCTA............GCCATGACAAGG........AGTTTTAAAT:1869

. . .2170 . . .2180 . . .2190 . . .2200 . . .2210 . . .2220

ASGH1 2062:TGTATTACAAATGGCTGTGATTGGGAGTCCCATAGGGCGACACGCAATTGGCCCACCGTC:2121

CSGH1 2065:TGTATTTCAAACGGCTGTGATTGTGAGTCCCATAGGGCGGCACACAATTCTCCCAGCGTC:2124

CSGH2 1861:TGTA....................................CACTTAAA......ATCAGC:1878

ASGH2 1870:TGTA....................................CACTTAAA......ATCAGC:1887

. . .2230 . . .2240 . . .2250 . . .2260 . . .2270 . . .2280

ASGH1 2122:GTTAGGGTTTGGCCGGGGTTGGCGGTCAAATAAAAAAAAATGGTGGAAATGAAATCTAGC:2181

CSGH1 2125:GTTAGGGTTTGGCCGGGGTTG.......................................:2145

CSGH2 1879:AGTAAAAT.........GTTG.......................................:1890

ASGH2 1888:AGTAAAAT.........GTTG.......................................:1899

. . .2290 . . .2300 . . .2310 . . .2320 . . .2330 . . .2340

ASGH1 2182:CATGACAGAGAGTTTAACTGTACATGTAAAATTGGCATTAACACATTGCTATACCTCAGT:2241

CSGH1 2145:................................................CAATACCTCAGT:2157

CSGH2 1890:................................................CTATACCTCAGT:1902

ASGH2 1899:................................................CTATACCTCAGT:1911

. . .2350 . . .2360 . . .2370 . . .2380 . . .2390 . . .2400

ASGH1 2242:GCCTTCAACTAAGGTAGGTAAAA.CAACCACATATCAAAGTCATTGCAAGTAAAACC.AT:2299

CSGH1 2158:GTCTTCAACTAAGGTAGATAAAA.CAACCACATAT......CATTGCAAGTAAAACC.AT:2209

CSGH2 1903:GCCTTCAACTAAGGTAGGTAAAAACAACCACATATCACAGTCCTTGTAAGTAAAACCCAT:1962

ASGH2 1912:GCTTTCAACTAAGGTAGGTAAAA.CAACCACATATCACAGTCCTTGTAAGTAAAACC.AT:1969

. . .2410 . . .2420 . . .2430 . . .2440 . . .2450 . . .2460

ASGH1 2300:CACTCTCTAAATCGGTGGTTTCTCTACGTCTACATTCTCCGTTTTGTGCTTTTCTGTCCA:2359

CSGH1 2210:CACTGTCTAA.TCGGTGGTTTCTCTATGTCTACATTCTCTGTTTTGTGCTTTTCTGTACA:2268

CSGH2 1963:CACTCTCTAA.TCGGCGGTTTCTCTACGTCTACATTCTC.....................:2000

ASGH2 1970:CACTCTCTAA.TCGGCGATTTCTCTACGTCTACATTCTC.....................:2007

. . .2470 . . .2480 . . .2490 . . .2500 . . .2510 . . .2520

ASGH1 2360:GGAAACCAGCCCCAAAGGTTTTTAACTCAATCATGTAAATAGGGAATCTCAAGCTGTACA:2419

CSGH1 2269:GGAAACCCACCCCAAAAGTATTTCACTCAATCATGTAAATAGGGCATCTCAAGCTGTA.A:2327

CSGH2 2000:............CAGCAATGTATCATGTAA..ATG...ATATGGCATCTCAAGCTGTACA:2043

ASGH2 2007:............CAGCCATGTATCATGTAA..ATG...ATATGGCATCTCAAGCTGTACA:2050

. . .2530 . . .2540 . . .2550 . . .2560 . . .2570 . . .2580

ASGH1 2420:AT.ACAACGCAACTTCATTTTCCAATAATCTGTGGTTTCTCTACATCTACACACACACAC:2478

CSGH1 2328:AT.ACAACTCAACTTCATTTTCCAATAATCTGTGGTTTCTCTACATCTTCACAC....AC:2382

CSGH2 2044:ATTACAACTCAACTTCATTTTCTAATCATCTGTGGTTTCTCTACATCTACACAC....AC:2099

ASGH2 2051:AT.ACAACTCAACTTCATTTTCTAATAATCTGTGGTTTCTCTGCATCTACACAC....AC:2105

. . .2590 . . .2600 . . .2610 . . .2620 . . .2630 . . .2640

ASGH1 2479:AGGTCCTGAAGCTGCTCCATATCTCTTTCCGTCTGATTGAATCCTGGGAGTACCCTAGCC:2538

CSGH1 2383:AGGTCCTGAAGCTGCTCCATATTTCTTTCCGTCTGATTGAATCCTGGGAGTACCCTAGCC:2442

CSGH2 2100:AGGTCCTGAAGCTGCTCCATATCTCTTTCCGCCTGATTGAATCCTGGGAGTACCCTAGCC:2159

ASGH2 2106:AGGTCCTGAAGCTGCTCCATATCTCTTTCCGCCTGATTGAATCCTGGGAGTACCCTAGCC:2165

Exon 4

. . .2650 . . .2660 . . .2670 . . .2680 . . .2690 . . .2700

ASGH1 2539:AGACCCTGACCATCTCCAACAGCCTAATGGTCAGAAACTCCAACCAGATCTCTGAGAAGC:2598

CSGH1 2443:AGACCCTGATCATCTCCAACAGCCTAATGGTCAGAAACGCCAACCAGATCTCTGAGAAGC:2502

CSGH2 2160:AGACCCTGACCATCTCCAACAGCCTAATGGTCAGAAACTACAACCAGATCTCCGAGAAGC:2219

ASGH2 2166:AGACCCTGGCCATCTCCAACAGCCTAATGGTCAGAAACTCCAACCAGATCTCTGAGAAGC:2225

. . .2710 . . .2720 . . .2730 . . .2740 . . .2750 . . .2760

ASGH1 2599:TCAGCGACCTCAAAGTGGGCATCAACCTGCTCATCAAGGTAAAGAAAGGAGGGAGAACAA:2658

CSGH1 2503:TCAGCGACCTCAAAGTGGGCATCAACCTGCTCATCACGGTAAATAATGGAGAGAGAACAA:2562

CSGH2 2220:TCAGCGACCTCAAAGTGGGCATCAACCTGCTCATCAAGGTAAT...........GGTCAA:2268

ASGH2 2226:TCAGCGACCTCAAAGTGGGCATCAATCTGCTCATCAAGGTAAAGAAAGGAGGGAGAACAA:2285

. . .2770 . . .2780 . . .2790 . . .2800 . . .2810 . . .2820

ASGH1 2659:TGACCATTTGTGGTGCCACACTTTGTGCACTGTAAACCCCAAGGCATTTTTAACTCAAAT:2718

CSGH1 2563:TGACCATTTGTGGTCTCACACTTTGTGCACTGTAAACTCCAAGGCATTTTTAACTCAAAT:2622

CSGH2 2269:TTACCATTTGTGGTGCCGCACTTTGTGCA.................TTTTTAACTCAAAT:2311

ASGH2 2286:TGACCATTTGTGGTGCCGCACTTTGTGCACTGTAAACCACAAGGCATTTTTAACTCAAAT:2345

. . .2830 . . .2840 . . .2850 . . .2860 . . .2870 . . .2880

ASGH1 2719:ACTTCTAGTAAGTTGAACTCAAAGTCAATGAAAAGTCATTATTACTTAAAATGTTTATGT:2778

CSGH1 2623:ACTTCTAGTAAGTTGAACTCAAGGTCAATGAAAAATCCTTATTGCTTAAAATGTTTATGT:2682

CSGH2 2312:ACTTCTAGTAAGTTGAAGTCA..GTCAATGAAAAGTCATTATTACTTCAAATGTCTATGT:2369

ASGH2 2346:ACTTCTAGTAAGTTGAACTCA..GTCAATGAAAAGTCATTATTACTTAAAATGTCTATGT:2403

. . .2890 . . .2900 . . .2910 . . .2920 . . .2930 . . .2940

ASGH1 2779:GGTACTGGCTCAAAACTAAATGAGAAGTGACATCAACACAATTTTTTAAAGTTATAACAA:2838

CSGH1 2683:GGTACTGGCTCAAAACTAAATGAGAAGTCACATCAATGCAATTTTTTTAAGTTATAACAA:2742

CSGH2 2370:GGTACTGGCTCAAATCTAAATGAG...TCACATCAATGCAATTTTTTAAAGTTATAACAA:2426

ASGH2 2404:GGTACTGGCTCAAATCTAAATGAG...TCACATTAATGCAATTTTTTTAAGTTATAACAA:2460

. . .2950 . . .2960 . . .2970 . . .2980 . . .2990 . . .3000

ASGH1 2839:ATTAACTTTTTATCCAGCATGCTCTACTGCAGGTAGATTTTTTGGAATT...........:2887

CSGH1 2743:ATTCACTTTT.ACCAAGCATGCTCTACTGCAGGTAGATTTAAAAAAAAAAATAAATAAAA:2801

CSGH2 2427:ATGAACTTTTTACCCAGCATGCTCTACTACAGGTAGATTTTTTGGAATT...........:2475

ASGH2 2461:ATTAACTTTTTACCCAGCATGCTCTACTACAGGTATATTTTTTGGAATT...........:2509

. . .3010 . . .3020 . . .3030 . . .3040 . . .3050 . . .3060

ASGH1 2887:...............GTTTTTAACTATCTGTGTTTT.GCATGTACAGA..........AC:2921

CSGH1 2802:AAAAAAAAAAAAGAAGTTTTTAATGATCTGTGTTTTTGCATGTACAGA..........AC:2851

CSGH2 2475:...............GTTTTTAAT.ATCTGTGTTTTTGCATGTACAGT..........AC:2509

ASGH2 2509:...............GTTTTTAAT.ATCTGTGTTTTTGCATGTACTATTTGCATTTTTGC:2553

. . .3070 . . .3080 . . .3090 . . .3100 . . .3110 . . .3120

ASGH1 2922:ATTGAGTGGTTGATTCATC....GTATGCTACACAAAGATATATAACATACATTTTTCAA:2977

CSGH1 2852:ATTGAGTGATTGATTCATT....TTATGCTACACAAAGATATATAACATACATGTTTCAA:2907

CSGH2 2510:ATTGAGTGATTGATTGATC....TTATGCTACACACAGATATATAACGTACATTTTTCTA:2565

ASGH2 2554:ATTGAGTGATTGATTGATTAATTTTATGCTTCACACAGATATATAACATACATTTTTCTA:2613

**(GATT)_n_ microsatellite**

. . .3130 . . .3140 . . .3150 . . .3160 . . .3170 . . .3180

ASGH1 2978:CATTTTCACAAAGATGAA.......TAAGTTACCAGAATTTTGCAAACCCGACTTGCAGG:3030

CSGH1 2908:CGTTTTCATAAAGATGAA.......CAAGTTACTAGAATTTTGCAAACTCAACTTGCAGG:2960

CSGH2 2566:CATTTTCACAAAGATAAATAACATACAAGGTACCGGAATTTTGCAAACCT.ACTTGCAGG:2624

ASGH2 2614:CGTTTTCACAAAGATAAA.......TAACATACCGGAATTTTGCAAACCT.ACTTGCAGG:2665

. . .3190 . . .3200 . . .3210 . . .3220 . . .3230 . . .3240

ASGH1 3031:CCTGATGTGGCCTGTAAACTATGAGTTTCAGGCCACTGTATTAGGGTACAGCTACGCCTC:3090

CSGH1 2961:CATGATGTGGCCTGTATACCGTGAGTTTCAGGCCACTGTATTAGGGTAAAGCTACGCCTC:3020

CSGH2 2625:CCTGATGTGGCCTGTAAACCATGAGTTTCAGGCCACTGTATTTGGGTAAAGCTACACCTC:2684

ASGH2 2666:CCTGATGTGGCCTGTAAACCATGAGTTTCAGGCCACTGTATTAGGGTAAAGCTACACCTC:2725

. . .3250 . . .3260 . . .3270 . . .3280 . . .3290 . . .3300

ASGH1 3091:AAAATACGGTCTTATGAGATATGTAATGTATTGTTATAAAGAGTTGAATTACAATGATAA:3150

CSGH1 3021:AAAATAAGGTCTTATGAGATAAGTAATGTATTGTTGTAAAGAGTTGAATTCTAATGATAA:3080

CSGH2 2685:AAAAGAAGGCCTTATAAGATATGTAATATATTGTTATAAAGAGTTTAACTATAATGATAA:2744

ASGH2 2726:AAAATAAGGCCTTATGAGATATGTAATATATTGTTATAAAGAGTTTAACTATAATGATAA:2785

. . .3310 . . .3320 . . .3330 . . .3340 . . .3350 . . .3360

ASGH1 3151:TATTTGCCTAGGAATTAACTTGAAGGCCACAGGACTGAAAATGAATGACAAC...CATGT:3207

CSGH1 3081:TATTTGCCTAGGAATTCACTTGAAGGCCACAGGACTGAAAATGAATGACAACAGCCATGT:3140

CSGH2 2745:TATTTGCCTGGAAAATCACTTGAAGGCCACAGGACTGAAAATTAATGACAACAAACATG.:2803

ASGH2 2786:TATTTGCCTAGAAAATCACTTGATGGCCACAGGACTGAAAATGAATGACAACAAACATGT:2845

. . .3370 . . .3380 . . .3390 . . .3400 . . .3410 . . .3420

ASGH1 3208:CTCTGTTACTAACAAATACAGTCATGGGTGATAACT..ACAATTCACTCAAAAAGGCCAG:3265

CSGH1 3141:CTCTGTCACTAACACATACAGTCATGGGTGATAACT..ACACTTTACTCAAAAAGGCCAG:3198

CSGH2 2803:....ATAACT........................CT..ACAATTCACTCAAAA.GGCAAG:2832

ASGH2 2846:CTCTGTCGCTAACAAATACAGTCATGGGTGATAACTCGACAATTCACTCAAAA.GGCAAG:2904

. . .3430 . . .3440 . . .3450 . . .3460 . . .3470 . . .3480

ASGH1 3266:GCACACTGGGAAATGATATTGGGGACGTGGCTTAGTGAGGGCATTACTAAAAAATGTCAA:3325

CSGH1 3199:GCACACTGGGAAATTATATTTGAGACGTGGCTTAGTGGGGGCATTACTAAAAAATGTCAA:3258

CSGH2 2833:GCACACTTGGAAATTATATTGGAGACATGGCTTAGTGGGGGCATTACTAAAAAATGTCAA:2892

ASGH2 2905:GCACACTTGGAAATTATATTGGAGACATGGCTTAGTGCGGGCATTACTAATAAATGTCAA:2964

. . .3490 . . .3500 . . .3510 . . .3520 . . .3530 . . .3540

ASGH1 3326:GCTGATACAACTCAAATCTGGACCCTTCACAGGGTGA.........CTAGAGTAATGACT:3376

CSGH1 3259:GCTGATACAACTCAAATCTGGACTCATCACAGGGTGAATCTATAGGTTTGAGTAATTACT:3318

CSGH2 2893:GCTGATACCACTCAAATCTCAACCCTCTACAGGGCGACTCTATAGGTTTGAGTAATGACT:2952

ASGH2 2965:GCTGATACCACTCAAATCTCAAC.CTCTACAGGGTGACTCTATAGGTTTGAGTAATGACT:3023

. . .3550 . . .3560 . . .3570 . . .3580 . . .3590 . . .3600

ASGH1 3376:.....................AACTGCAGTCAGATTCTATATATTAAGTGCAACGGGTTT:3415

CSGH1 3319:GACTATAATATCACTTTAAGTAACTGCAGTCAGATTCTGTATATTAAGTGCAACGGGTTT:3378

CSGH2 2953:ATA...AAAATCACTTTAAATGACTGTAGTCAGATTCTGTATATTAAGTGCAACGG.TTT:3008

ASGH2 3024:ATA...AAAATCACTTTAAGTAACTGTAGTCAGATTCTGTTTATTAAGTGCAACGG.TTT:3079

**CRE**

. . .3610 . . .3620 . . .3630 . . .3640 . . .3650 . . .3660

ASGH1 3416:CCTAAAACGTTTTGAGTAATGACAGCACATTGGGTTTTACAGTG................:3459

CSGH1 3379:CCTAAAAAGTGTTGAGTAATGGCAGCACATTGGGGTTTACAGTG................:3422

CSGH2 3009:CCTCAAAAGTTTTGAGTAATGACAGCACATTGGGGTTTACAATGTGGTTATTAACTTCCA:3068

ASGH2 3080:CCTCAAAAGTTTTGAGTAATGACAGCACATTGGGGTTTACAGTGTGGTTATTATCTTCCA:3139

. . .3670 . . .3680 . . .3690 . . .3700 . . .3710 . . .3720

ASGH1 3459:...ACATGAAAGTGAAATACCTCTATGCTTTCCTAGTTAGAAAGCATAGTGTA.GGACCA:3515

CSGH1 3422:...ACATGAAAGGGAAATACCTGTATGCTTTCCTAGTTAGAAAGCATAGTGTAAGGACCA:3479

CSGH2 3069:CTGACATGAAAGTGAAATACAACTATGCTTTCCTAGTTAGAAAGCATAGTGTA.GGACCA:3127

ASGH2 3140:CTGACATGAAAGTGAAATACAACTATGCTTTCCTAGTTAGAAAGCATAGTGTA.GGACTA:3198

. . .3730 . . .3740 . . .3750 . . .3760 . . .3770 . . .3780

ASGH1 3516:CGTTTGCCTCTTCTCAGCAGATCTTTCAGTGCTTTACATTGTGATGGGGTAAATAACCTC:3575

CSGH1 3480:CGTATGCCTCTTCTCAGCAGATCTTTCAGGGCTTTACATTGTGATGTGGTAACTGACCTT:3539

CSGH2 3128:CGTATGCCTCTTCTCAGCAGATCTTTCAGCGCTTTACATTGTGATGGGGTAACTCACCTC:3187

ASGH2 3199:CGTACGCCTCTTCTCAGCAGATCTTTCAGTGCTTTACATTGTGATGTGGTAACTCACCTC:3258

. . .3790 . . .3800 . . .3810 . . .3820 . . .3830 . . .3840

ASGH1 3576:ATCTATC..ATCACTAATATTGACTATATCAGTAACACCCCATTCAATGACTGAATATCA:3633

CSGH1 3540:ATCTATC..ATCG.......TGATTATATCAGTGACACCCCATTCAATGACTGAATATCG:3590

CSGH2 3188:ATTGATC..ATCACTAATAGTGACTATATCAGTAACACCCCATTCAATGACTGAATATTG:3245

ASGH2 3259:ATATATATAGTCACTAATAGTGACTATATCAGTAACACCCCATTCAATGACTGAATATTG:3318

. . .3850 . . .3860 . . .3870 . . .3880 . . .3890 . . .3900

ASGH1 3634:GCCCATTCAAGGATATTTATGCATGCGTCTTTTGCTATGTGTGCTTTCAGAAAGGCCCAA:3693

CSGH1 3591:CCCCATTCAAGGACATTTATCCATGTGTCTTTTGCTACGTGTGCTTTCAGAAAGGCCCAA:3650

CSGH2 3246:GCCCATTCAATGACATTTATGCATGTGTCTTTTGCTATATGTGCTTGTAGAATGGCCAAA:3305

ASGH2 3319:TCCCATTCAAGGACATCTATGCATG..TCTTTTGCTATATGTGCTTTTAGAATGGCCCAA:3376

. . .3910 . . .3920 . . .3930 . . .3940 . . .3950 . . .3960

ASGH1 3694:TAAACAAATATTGATATGCACACATCCACCCCACCATGCATCTCTCTCTGTCTCCCACAG:3753

CSGH1 3651:TAAACAAATATTGATATGCACACATCCACCCCACCATGCATCTCTCTCTGTCTCCCACAG:3710

CSGH2 3306:TAAACGAGTATTGATATGCACACATCCACCCCACCATGCATCTCTCTCTGTCTCCCACAG:3365

ASGH2 3377:TAAACAAATATTGATATGCACGCATCCACCCCACCATGCATCTCTCTCTGTCTCCCACAG:3436

. . .3970 . . .3980 . . .3990 . . .4000 . . .4010 . . .4020

ASGH1 3754:GGGAGCCAGGATGGCGTACTGAGCCTGGATGACAATGACTCTCAGCAGCTGCCCCCCTAC:3813

CSGH1 3711:GGGAGCCAGGATGGCCTACTGAGCCTGGATGACAATGACTCTCAGCAACTGCCCCCCTAC:3770

CSGH2 3366:GGGAGCCAGGATGGCGTACTGAGCCTGGATGACAATGACTCTCTGCATCTGCCCCCCTAC:3425

ASGH2 3437:GGGAGCCAGGATGGCGTACTGAGCCTGGATGACAATGACTCTCAGCATCTGCCTCCCTAC:3496

Exon 5

. . .4030 . . .4040 . . .4050 . . .4060 . . .4070 . . .4080

ASGH1 3814:GGGAACTACTACCAGAACCTGGGGGGCGACGGCAACGTCAGGAGGAACTATGAGTTGTTG:3873

CSGH1 3771:GGGAACTACTACCAGAACCTGGGGGGTGACGGAAACGTCAGGAGGAACTACGAGTTGTTG:3830

CSGH2 3426:GGGAACTACTACCAGAACCTGGGGGGCGACGGCAACGTCAGGAGGAACTACGAACTGTTG:3485

ASGH2 3497:GGGAACTACTACCAGAACCTGGGGGGCGATGGCAACATCAGGAGAAACTACGAACTGTTG:3556

. . .4090 . . .4100 . . .4110 . . .4120 . . .4130 . . .4140

ASGH1 3874:GCCTGCTTCAAGAAGGACATGCACAAGGTGCAAAACCATGTTGCCTTCTATTTCATGTGC:3933

CSGH1 3831:GCTTGCTTCAAGAAGGACATGCACAAGGTGCGCAACCATGTTGCCTTCAATTTTATGTGC:3890

CSGH2 3486:GCCTGCTTCAAGAAGGACATGCATAAGGTGGAAGATCATGTTGCCTTCAATTGCATGTGC:3545

ASGH2 3557:GCCTGCTTCAAGAAGGACATGCATAAGGTGGAAGACCATTTTGCCTTCAATTGCATGTGC:3616

. . .4150 . . .4160 . . .4170 . . .4180 . . .4190 . . .4200

ASGH1 3934:CTTCCTATATTTTCTACAGTGCGTT.....TCTTGTGCTCTCTATTGCAAAGTAT.....:3983

CSGH1 3891:CTTCCTGTATTTTCTACAGTGCGTTGTTT.TTTTGTATTCTCTATTGCAAAGTATTGTTA:3949

CSGH2 3546:CTTCCTATATTTTCTACGGTGCATTGTTTTTTTTGTAATCTCTATTGTGAAGTAT.....:3600

ASGH2 3617:CTTCCTATATTTTCTACAGTGAATTGTTT.TTTTGTGTTCTCTGTTGTGAAGTAT.....:3670

. . .4210 . . .4220 . . .4230 . . .4240 . . .4250 . . .4260

ASGH1 3983:............................................................:3983

CSGH1 3950:GTAAATAACTCACAGACACTAGAGAAGCTTTAACCAAGTTTAATTCTTCCCAAAGGTTCT:4009

CSGH2 3600:............................................................:3600

ASGH2 3670:............................................................:3670

. . .4270 . . .4280 . . .4290 . . .4300 . . .4310 . . .4320

ASGH1 3983:............................................................:3983

CSGH1 4010:GTACAGCTATAATCAGACAGCAAAACATTTCTCACTCCACAGTCATATATATCCTACTTA:4069

CSGH2 3600:............................................................:3600

ASGH2 3670:............................................................:3670

. . .4330 . . .4340 . . .4350 . . .4360 . . .4370 . . .4380

ASGH1 3983:............................................................:3983

CSGH1 4070:AAACACTCCTCCTTCTTCAATCCTTACAGTTTATGGCTCCACAGGAAGCTAATAAAGAGG:4129

CSGH2 3600:............................................................:3600

ASGH2 3670:............................................................:3670

. . .4390 . . .4400 . . .4410 . . .4420 . . .4430 . . .4440

ASGH1 3983:............................................................:3983

CSGH1 4130:GTAACAGGACAACAAACCTTTATTACTGCCTTCAGAGAATCTGTCCTCACCTCCTGACCT:4189

CSGH2 3600:............................................................:3600

ASGH2 3670:............................................................:3670

. . .4450 . . .4460 . . .4470 . . .4480 . . .4490 . . .4500

ASGH1 3983:............................................................:3983

CSGH1 4190:CGACCCCTCATCTAATCCACAGATGTCCATTGTTTTTTTTTCAGAGAACCATTAAGTTCT:4249

CSGH2 3600:............................................................:3600

ASGH2 3670:............................................................:3670

. . .4510 . . .4520 . . .4530 . . .4540 . . .4550 . . .4560

ASGH1 3983:............................................................:3983

CSGH1 4250:GACATAACCCAGTTTCTTTCATTTACTATCTCAATGATCAACGTTTAGCCGATTCCAACA:4309

CSGH2 3600:............................................................:3600

ASGH2 3670:............................................................:3670

. . .4570 . . .4580 . . .4590 . . .4600 . . .4610 . . .4620

ASGH1 3983:....CTTTGGGTCTTTAACCCATATATTATTACTATTATTGTTCATTGATCAAGACTGTT:4039

CSGH1 4310:GTATCTTTGGGTCTTTAACCCATGTATTATTACTATTATTGTTCATTGATCAAGACTGTT:4369

CSGH2 3600:....CTTTGGGTCTTCAACCCATATGTTATTACTATTATTGTTTATTGATCAAGACTGGT:3656

ASGH2 3670:....CTTTGGGTCTTTAACCCATATATTGTTACTATTATTGTTCATTGATCAAGACTGGC:3726

. . .4630 . . .4640 . . .4650 . . .4660 . . .4670 . . .4680

ASGH1 4040:CTCGAGAAAGGTCTAGTGACCTAGAACACTCACATTAAAATGTGTCAACTATAACCCATT:4099

CSGH1 4370:CTTGAGAAAGGTCTGGTGACCTAGAACACACACATTAAAATGTGTCAACTATAACCCATT:4429

CSGH2 3657:CTCGAGAAAGTCCTGGTGACTTAGAACATGCACATTAAAATGTGTCA.CTATAACCTATT:3715

ASGH2 3727:CTTGAGAAAGTCCTAGTGACTTAGAACATTCACATTAAAATGTGTCAACTATAACCTATT:3786

. . .4690 . . .4700 . . .4710 . . .4720 . . .4730 . . .4740

ASGH1 4100:CTTCTATTTTTCCC.CCAAGGTCGAGACCTACCTGACCGTCGCCAAGTGCAGGAAGTCAC:4158

CSGH1 4430:CTTCTTTTTTCCCCCCCGAGGTCGAGACCTACCTGACCGTCGCCAAGTGCAGGAAGTCAC:4489

CSGH2 3716:CTTCTTGT......CCCAAGGTTGAGACCTACCTGACCGTCGCTAAGTGCAGGAAGTCAC:3769

ASGH2 3787:CTTATTTTTTTC..CCCAAGGTTGAGACCTACCTGACCGTCGCTAAGTGCAGGAAGTCGC:3844

Exon 6

. . .4750 . . .4760 . . .4770 . . .4780 . . .4790 . . .4800

ASGH1 4159:TGGAGGCCAACTGCACTCTGTAGACGTGGGCTGGAGAGGCAGCCAGCAAGAGCCTGTCTC:4218

CSGH1 4490:TGGAGGCCAACTGCACTCTGTAGACGTGGGCTGGAGAGGCAGCCAGCAAGAGCCTATCTC:4549

CSGH2 3770:TGGAGGCCAACTGCACTCTGTAAACGTGGGCCGGAGCGGCAGCCAGCAAGAGCCTGTCTC:3829

ASGH2 3845:TGGAGGCCAACTGCACTCTGTAAACATGGGCTGGAGCGGAAGCCAGCAAGAGCCTGTCTC:3904

>>> 3’-untranslated region >>>

. . .4810 . . .4820 . . .4830 . . .4840 . . .4850 . . .4860

ASGH1 4219:CAGGGTTCGGTTTCCCAGATACAGATTAGGCCTTGCCCTGCACTGAAGAGCATTTTCAAT:4278

CSGH1 4550:CAGGGTTCGGTTTCCCAGATACAGATTAGGCCTTGCCCTGCACTGAGGTGCATTTTCAAT:4609

CSGH2 3830:CAGGGTTCGGTTCCCCAGATACAGATGAGACCTTGCCCTGCACTGAAGAGCATGTTCAAT:3889

ASGH2 3905:CAGGGTTAGGTTTCCCAGATACAGATTAGACCTTGCCCTGCACTGAAGTGCATTTTCAAT:3964

. . .4870 . . .4880 . . .4890 . . .4900 . . .4910 . . .4920

ASGH1 4279:TGAGATTCTCCATTAAACGTGCTTTTT.AGTCTTGAGTAGATTTAATTTGGATCTGGTAG:4337

CSGH1 4610:TGAGATTCTCCATTAAACATGCTTTTC.AGTCTAGAGTAATTTTATTTTGGATCTGGTAG:4668

CSGH2 3890:TGAGATTCTCTATTAGGCATGTTTTTTTAGTCTAGAGTAGATTTCATTTGGATCTGGTAG:3949

ASGH2 3965:TGAGATTCTCCATTAGGCATGCTTTTT.AGTCTAGAGTAGATTTAATTTGGATCTGGTAG:4023

. . .4930 . . .4940 . . .4950 . . .4960 . . .4970 . . .4980

ASGH1 4338:AGCCTGACTCCAGGGGTTTTCAGGAATTT.GCATTTTGTTCTCTGAAATCAACAACAGCA:4396

CSGH1 4669:AGCCTGACTCCAGGGGTTTTCAGGCATTT.GCATTTTTTTCTCTGAAATCAATAACAACA:4727

CSGH2 3950:AGCCTGGCTCCAGGGGTTTTCAAGCATTTTGCATTTTGTTCTCTGAAATCAAC.....TT:4004

ASGH2 4024:AGCCTGACTCCAGGGGTTTTCAAGCATTT.GCATTTTGTTCTCTGAAATCAACAACAGCA:4082

. . .4990 . . .5000 . . .5010 . . .5020 . . .5030 . . .5040

ASGH1 4397:CTTTCTAT...ATTGACT..ATTACTCTGAGCTACCATTGAT.................T:4434

CSGH1 4728:CTTTCTAT...ATTGACTCTATCACTCTGAGCTACCATTGAT.................T:4767

CSGH2 4005:TCTATGAT...ATTCACTCCATTACTCGGAGCTACCACTGATCCATGGACATTTTAGATT:4061

ASGH2 4083:CTTTCTATGATATTCACTCTATTACTCATTGAT.CTATAGAC........ATTTTAGATT:4133

. . .5050 . . .5060 . . .5070 . . .5080 . . .5090 . . .5100

ASGH1 4435:AGTACATTTATAGAAAAGGTTATTAAAT..GTCTTATTTAGATATATGATTCATGGTGGT:4492

CSGH1 4768:AGTACATTTATATTAAAGGTTATTAAAT..GTCTTATTTAGATATATGGTTCATGGCGGT:4825

CSGH2 4062:AGTACATTTATAGAAACGGTTTATAAATATGTCTTATTTAGATATATGATTCAAGGTGGT:4121

ASGH2 4134:AGTACATTTATAGAAAAGTTTATAAAAA.CGTGTTATTTAGATATATGATTCAAGGTGGT:4192

. . .5110 . . .5120 . . .5130 . . .5140 . . .5150 . . .5160

ASGH1 4493:GCTACTGTTTATGCATACATTCATATTTAGGGGTGAAATGGGAACGTGTAGAGCTCCAAG:4552

CSGH1 4826:GCTAC...TTATGCATACGTTAATATTTAGGGGTGAAATGGGAACTTGTAGAGCTCCAAG:4882

CSGH2 4122:GGTGCCATTTATGTATACATTAATATTTAGGGGTGAAATGGGAACATGTAGAGCTCCAAT:4181

ASGH2 4193:GCTGCAATTTATGCATACATTAATATTTAGGGGTGAAATGGGAACTTGTAGAGCTCCAAG:4252

. . .5170 . . .5180 . . .5190 . . .5200 . . .5210 . . .5220

ASGH1 4553:CTTT...............TGGATAATATATTTTAGAGTTATTTCCTTTAAGTATTTTCA:4597

CSGH1 4883:CTTT...............TGGATAATATATTTTAGAGTAATTTCCTTTAAGTATTTTCA:4927

CSGH2 4182:CTTTAGGTATGTCCACAGATGGATAATATATTTTAGAGTCATTTCCTTGAAGTATTTTCA:4241

ASGH2 4253:CTTTTGGTATGTCCACAGATGGATAATATATTTTAGAGTAATTTCATTTAAGTATTTTCA:4312

. . .5230 . . .5240 . . .5250 . . .5260 . . .5270 . . .5280

ASGH1 4598:TTTCTTAATCTTATTGTTTGAAACTAATAGTGATACATTTTTCAATAAAGCTGTTGTTCT:4657

CSGH1 4928:TTCCTTAATCTTATTGTTTGAAACTAATAGTGATTCATGTTTCAATAAAGATGTTCTTCT:4987

CSGH2 4242:TTCCTTTATCTTACTGTTGGAAACGAATAGTGATTTGT.TTTCAATAAA......CTTCT:4294

ASGH2 4313:TTCCTTAATCTTACTGTTTGAAACTAATAGTGATTAGT.TTTCAATAAAGCTGTTGTTCT:4371

**Poly(A)termination signal;1**

. . .5290 . . .5300 . . .5310 . . .5320 . . .5330 . . .5340

ASGH1 4658:CTGCAGTACATGATCTCTTGGCTACTATTTACTATCTTTCAAATCAACATTTTTT..ACA:4715

CSGH1 4988:CTGCAGCACATGATCTCTTGGCTTCTATTTAATATCTTTCAAATCAACATTTTTT..ACA:5045

CSGH2 4295:CTGCGGTACATGATCTCTTGGCTACTATTTGCTATCTTTCAAATCAATTTTTTTTTTACA:4354

ASGH2 4372:CTGCGGTACATGATCTCTTGGCTACTATTTGCTATCTTTCAAATCAACATTTTTT..ACA:4429

. . .5350 . . .5360 . . .5370 . . .5380 . . .5390 . . .5400

ASGH1 4716:AGTTCCTAGCCCCAACATTCCTATGGTGTCCCTTGGACAATTTAGGGCTGGATTCAATCC:4775

CSGH1 5046:AGTTCCTAGCCACAACATTCCTATGGTGTCTCTCGGACA.......GCTGGATTCAATCC:5098

CSGH2 4355:AGTTTCTAGCCCCAGCATTCCTATTGTGTCCCTTGGACAACTTAAGGCTGGATTCAATTC:4414

ASGH2 4430:AGTTTCTAGCCCCAACATTCCTATTGTGTCCCTTGGACAACTTAAGGCTGGATTCAATTC:4489

. . .5410 . . .5420 . . .5430 . . .5440 . . .5450 . . .5460

ASGH1 4776:GTATCGCAGGCACTCCATTGAAATGTAAAGGCAATGTTCC.TGCGTTCGCGGAGACTGCA:4834

CSGH1 5099:GTATCGCAGACGCTCCATTGAAATGTAAAGGCAATGTTCC.TGCGTTCGCGGAGACTGCA:5157

CSGH2 4415:GTATCGCAGACGCTCCATTGAAATGTAAAGGCAATGTTCCCTGCGTTCGTGGAGACGGCA:4474

ASGH2 4490:GTATCACAGACGCTCCATTGAA.TGTAAAGGCAATGTTCC.CGCGTTCGTGGAGACGGCA:4547

. . .5470 . . .5480 . . .5490 . . .5500 . . .5510 . . .5520

ASGH1 4835:TTCACT.........GCATATGTC.GGCTCAATCGG.AAATTACCTTAAAAATGTTCCGC:4883

CSGH1 5158:TTCACTTCAAACGCTGCATATGTC.GGCTCAATCGG.AAATTACCTGAAAAACGTTACAC:5215

CSGH2 4475:TTCACCGTAAACGCTGCATATGTCAGGCTCGTTCGGGAAATGACCTTAAAGATGTTACGC:4534

ASGH2 4548:TTCACCTGA.....TGAATCTGTT..GATCGTT.......TTCCTCCTAGAACGAGTCAA:4593

. . .5530 . . .5540 . . .5550 . . .5560 . . .5570 . . .5580

ASGH1 4884:GGTTCTTCAGCGATACGGATTGAATCCAGCCCATAGT.TACGTACATTTGAATTGGAAAA:4942

CSGH1 5216:GGTTCTTCAGCGATACGGATTGAATCAAGCCCATAGT.TACGTACATTTGTATTGGCAAA:5274

CSGH2 4535:TGTTCTTCGGCAATACAGATTGAAAACAGCCCATAGT.CACGTACATTCGTATTGTA...:4590

ASGH2 4594:AGC.CTTCAA.AATA.GCAATGTCCTCACTACATTATGCAATGATCTCTCTGTTGATGAT:4650

. . .5590 . . .5600 . . .5610 . . .5620 . . .5630 . . .5640

ASGH1 4943:AAACATGAATGTCCACTGTCTGTTGCGAATG..TTGAATAAAACTCAATTTGAACTTTTT:5000

CSGH1 5275:AA.CATGAATGTCCACCGTCTGTTGCGAATG..TTGAATAAAACTCCATTTGAACTTTGT:5331

CSGH2 4590:...CATCAATATCCCCTGTCTGTTGCGAATG..TTGAATAAAGCTCAATTTGAACTTTGT:4645

ASGH2 4651:GTCTGCCGTTATTTCAATCCGGATGCAAAGGGGTTACACCTGGACCAACAACAACTATGT:4710

**Poly(A)termination signal;2**

. . .5650 . . .5660 . . .5670 . . .5680 . . .5690 . . .5700

ASGH1 5001:C.TGCCGATTGTCCATAGGGTTGGTTGTTAAATACCTCGG..AATTTGAGAAA.AGAT.A:5055

CSGH1 5332:C.TGCCAATTGTCCATAGGGTTGGTTGTTATATACCTCGG..AATTTGAGAAA.AGAT.A:5386

CSGH2 4646:C.TGCTGATTGTCCATAGAGTTGGTTGTTATATAGCTCGGG.AATTTGAGAAA.AGAT.A:4701

ASGH2 4711:CACATAAATCTACAATATATTTATTCCTAATATCCCCCGTTTTGTTACAGATGTAGATCA:4770

. . .5710 . . .5720 . . .5730 . . .5740 . . .5750 . . .5760

ASGH1 5056:TCCGAAGGAACAT...AATAAA......CAGACTT.TCCAAACGTG...G..GTCTGCAG:5100

CSGH1 5387:TCCGATGGAACATATTAATAAA......CAGACTT.TCCAAACATG...G..GTCTGCTG:5434

CSGH2 4702:TCCAAAGGAACATACATACAAAGG....CTGGTCT.CCTGCAGGTGCCTA..ATATGTCA:4754

ASGH2 4771:TCAGTTGGCTCCCTTTCGTGATCATCACTTGATTTATCTGAATTTACAAGCTACTTGTAA:4830

**Poly(A)termination signal;3**

. . .5770 . . .5780 . . .5790 . . .5800 . . .5810 . . .5820

ASGH1 5101:CAG......GTGCCCGA...CACCATGCCCAA.ACTGGAAGC.GCACGTGC.....GCCA:5144

CSGH1 5435:CAG......GTGCCTAA...CACCATGCCCAG.ACTGGACGC.GCGCGTGC.....GCAA:5478

CSGH2 4755:AAACACCTCATGTCAAAACACCTCATGTCAAA.ACAAAAGTC.CTGCAAGT.....GCAA:4807

ASGH2 4831:TGAACATGAGGGGAGAAAGAGCTGATTTCAAGTGCAGGGCACAGCAGGTGTTTATTGCAA:4890

. . .5830 . . .5840 . . .5850 . . .5860 . . .5870 . . .5880

ASGH1 5145:TCGTGCGCACATTGATTTTGCCCCCCACCCCCACCCCAAACGCGATCA.CA.ACAC.GCA:5201

CSGH1 5478:..........ATTGATTTTGTCC....CCCCCACACCAAAGGCGATCA.CA.ACAC.GCA:5521

CSGH2 4808:GCAAGCGATAACTTGTGCCAGACTT.ATTTTGACAGAGAGGAAGATGGGTA.TTGCAGCA:4865

ASGH2 4891:AGGACCACAGGAGGAGGCAGGTAGCTGGGTCCAGGGGCAGGCAGAAGGTCATACACAGGG:4950

. . .5890 . . .5900 . . .5910 . . .5920 . . .5930 . . .5940

ASGH1 5202:GGTTGAAA....TATCAAAACAAATTCTGAACCAATT.ATATTGATTTGGGGATAGGTCA:5256

CSGH1 5522:GGTTGAAA....TATCAAAACAATCTCTGAACCAATT.ATATTCATTTGGGGATAGGTCG:5576

CSGH2 4866:GACCTCTT....TATTGACATGTTCCTTCAGATATTTTGTCTCAAATTCCACGCTATACA:4921

ASGH2 4951:GGTCCAAAAGGGCAACAGTACAGGCAGGGAAAAGGCTAGTAACGTAGTCCGGG.AGATCA:5009

. . .5950 . . .5960 . . .5970 . . .5980 . . .5990 . . .6000

ASGH1 5257:AAAAGCATTAAACATTTATGGCAATTT.AGCTAGCTAGCTTGCAGTTGCTAGCTA.ATTT:5314

CSGH1 5577:AAAAGCATTAAACATTTATGGCAATTT.AGTTAGCTAGCTTGCAGTTGCTAGCTA.ATTT:5634

CSGH2 4922:AAAACCAA.ACCCATGGACAATTGTCAGAGTAAGTTCAAATGGAATTTTATTCAACATTC:4980

ASGH2 5010:GGCAATAG.GTAGATGACAGGAAATCC.GATAGGCTAAAGTACAGCCAGGGAATAGGCAA:5067

. . .6010 . . .6020 . . .6030 . . .6040 . . .6050 . . .6060

ASGH1 5314:.TCCAAT..TTAGCTAGCTTGCTGTT.GCTAGCTAATTTGGGA..TATAAA.TGTTGAGT:5367

CSGH1 5635:GTCCCAT..TTAGCTAGCTTGCTGTT.GCTAGCTAATTTGGGA..TATAAA.TGTTGAGT:5688

CSGH2 4981:ATGACAC..ACAGTGGATGTGAACCTAGCTTTCAGGTTTTGGC..AATGGAGTTTTGCGC:5036

ASGH2 5068:AAGGCATCGTTAGTGAGGCAGGCGAAAACTATCATACACAGGAGGAGTAAATCATGGGAA:5127

. . .6070 . . .6080 . . .6090 . . .6100 . . .6110 . . .6120

ASGH1 5368:TGTTATTTTACCTGAAATTCACAAGGTCC....TCTACTCTGACAATTAA...TCCACAC:5420

CSGH1 5689:TGTTATTTTACCTGAAATTCACAAGGTCC....TCTACTCTGACAATTAA...TCCACAC:5741

CSGH2 5037:GCTGAGGATGTCAGAGGG..ATAGCGTCGG...TGCATCATGAATAACAAGT.TCCATGC:5090

ASGH2 5128:ACCCAGAGCTCCTGAAAGACGTGTGTCACAAAACAAACAATACCTCACAGTTATGGGGGT:5187

. . .6130 . . .6140 . . .6150 . . .6160 . . .6170 . . .6180

ASGH1 5421:ATAAACCAGTCAACCGAATCGTTTCCAGTCATCTCTCCTCCATCCAAGCTTTTTCTTCTT:5480

CSGH1 5742:ATAAAACAGTCAACCGAATTGTTTCCAGTCATCTCTCCTCCTTCCAGGCCTTTTCATCTT:5801

CSGH2 5091:ATTAAACAT...AATGAACATTTCAGTGTTATATGAAGTGAGTTGAGTCTTAGTAGTACC:5147

ASGH2 5188:GCAAAGAACTGAACTAAATAGTGTGTGATAATGACATATAGGTGTGTGAACAG..GTGAT:5245

. . .6190 . . .6200 . . .6210 . . .6220 . . .6230 . . .6240

ASGH1 5481:TGGATTCTATATGGC.ATTGGCAGCTAACT..TTCATAGTA.TTGCCACGACGACCGACC:5536

CSGH1 5802:TGGACTTTAAATGGCGATTGGCATCTAACA..TTCATAGTA.TTGCCACGACGACCGACT:5858

CSGH2 5148:AGAATCTTATATTTACAAAAGCACCACACCACCTCACATCAACTCCCACCATAACTAAAT:5207

ASGH2 5246:TAGAATTCAAGTG...ATTGGGATCTGGAGAGTGAGTTACGTTCAGGGGATCTAGGTGTT:5302

. . .6250 . . .6260 . . .6270 . . .6280 . . .6290 . . .6300

ASGH1 5537:GACCTACCTCAGTTCATCTTTCAATCACCCACGTGGGTATAACCAATGA..GGAGATGGC:5594

CSGH1 5859:GA....CCTCAGTTCATCTTTCAATAACCCACGTGGGTATAACCAATGA..GGTGATGGC:5912

CSGH2 5208:CTATGGACTTACCTTCAGTACTAGTAGCTGTGTTATAATTTACCATTCATCGTTAACGTC:5267

ASGH2 5303:TGAGAGTGTGAGCTGGAAAGTGGGCTGGAAAGTGAGCCGCATTCAGGGGATCTTCATGTT:5362

. . .6310 . . .6320 . . .6330 . . .6340 . . .6350 . . .6360

ASGH1 5594:.A....CGTGGGT.ATCTGCTTCTA.TAAACCAATGAGGAGATG.GGAAAGGCAGGACT.:5645

CSGH1 5912:.A....CGTGGGT.ATCTGCTTCTA.TTAACCAATGAGGAGATG.TGAGAGGCATGACT.:5963

CSGH2 5267:.AATGCCATGAAC.AGTGGCGTACAGTGGATTCACGAGGCTCCCCTGCAAGGTACGAGC.:5324

ASGH2 5363:TGAGGGTGTGAGTTGGAAGCAGACATTACACT.ACAAAAAAATCTAAAGGCACTCGAGGA:5421

. . .6370 . . .6380 . . .6390 . . .6400 . . .6410 . . .6420

ASGH1 5646:TGTACCGTATTCAACGTCAC........AAATAGAACTAACTTCTATTTTAGGGCTTGGC:5697

CSGH1 5964:TGCACCGTGTTCAATGTCAC........AAATAGAACTGACTTCTATTATAGGGCTTGGC:6015

CSGH2 5325:AAGACCAATTGCAAAGCCAGGGGGCGGGAAATAGAATGCGATGCTAATGTTAACATTTTT:5384

ASGH2 5422:TATTGGAAATTAAACAACACA...CTTCTTAAAGATCCTATTCTCATTGGAAACATCAAA:5478

. . .6430 . . .6440 . . .6450 . . .6460 . . .6470 . . .6480

ASGH1 5698:AACGCAG..ATGCTCG...TTGGCGAGCGCGAGCAGTGTGGGTGC.AATAATTGAATAAC:5751

CSGH1 6016:AACGCAG..ATGCTCA...TTGACGAGCGTGAGCAGTGTGGGTGC.AATATTTGAATAAC:6069

CSGH2 5385:ACCACAGGAACATTTGACCTTGTTGCCAACAGACAATTAGCCTACCAACCTCTGAATATA:5444

ASGH2 5479:TCGTTAGCCAAAGATATTTTTTGCAAGAGAAGACTTGGGTCATGGAAGTAGATGGGAATT:5538

. . .6490 . . .6500 . . .6510 . . .6520 . . .6530 . . .6540

ASGH1 5752:ATG.......TATGTATACATTTATT.TTGCAATGCTTGCGCATGCGACATGAT.AGGTG:5802

CSGH1 6070:ATG.......TATGTGTACATTTATT.TTCCAATGCTCGCGCACGCGACATGAG.CGGTG:6120

CSGH2 5445:TTGAGTCAATTATGGTCCTACAGGTA.TTTAAGGGACTTCAATTGTGAGCTAAAACGATT:5503

ASGH2 5539:CTT.......CAAATATAAAGTCAGAGTTGTAGCCATTAAACGTGCCAAA.GAGCTGATG:5590

. . .6550 . . .6560 . . .6570 . . .6580 . . .6590 . . .6600

ASGH1 5803:TGGTCAGCATGTAACATGTCAAACACC..TCATGTCAAAACAAAA.GTCCTGCAAGTGCG:5859

CSGH1 6121:TGGTCAGCATGTAACATGTCAAACACC..TCATGCCAAAACAAAA.GTCCTGCAAGTGCG:6177

CSGH2 5504:CGATTATTTTACTTTCCTCAGGTCTTCGATCATGGGCAACTCCAA.ACCCTTTGGTGGCC:5562

ASGH2 5591:CAATTAAAGAACCTTAGAGAAAAAGAG.ATGATGAGCAAGCTTGACAGTTTTCTAAAGAA:5649

. . .6610 . . .6620 . . .6630 . . .6640 . . .6650 . . .6660

ASGH1 5860:CCATATGTTGGCAAGCAAGCCATAACTTGTGCCAG.ACTTTTATTTTGACAGAG..GACG:5916

CSGH1 6178:CCATATGTTGGCAAGCAAGCCATAACTTGTGCCAG.ACTTTTATTTTGACAGAGAGGAAG:6236

CSGH2 5563:CCAACAAACTTTAACCAGATGAAGGTGTGTCCCAGCATTCGCAATTTCATTGGTTACGCA:5622

ASGH2 5650:AGATAATCTATCTCAAGAAGAAGAATCTGTATTTAAATCTTTACAGCTAGAACTAGAACA:5709

. . .6670 . . .6680 . . .6690 . . .6700 . . .6710 . . .6720

ASGH1 5917:AGGGTATGCAGCTGACCTGTTTATTGATATGTTCCTTCAGATATTTTGT.CTCAAATTCC:5975

CSGH1 6237:AGGGTATGCAGCTGACCTGTTTATTGATATGTTCCTTCAGATATTTTGT.CTCAAATTCC:6295

CSGH2 5623:GCTGTAAGCA.CTGCCCCGTGCTCGAGCTCATTGGTC...GTGTTGGGTACAGAAAATCT:5678

ASGH2 5710:GCTTTACACGGCT...CTGTACTATACAAAATCTAAAACTGTACTCAAA..ATGAATGAT:5764

. . .6730 . . .6740 . . .6750 . . .6760 . . .6770 . . .6780

ASGH1 5976:ACGCTATATATAAA..CCA..ACCCCATGGACAATCGTCAG..AGTAAGTTCAAATTGAA:6029

CSGH1 6296:ACGCTATGTATAAA..CCA..ACCCCATGGACAATCGTCAG..AGTAAGTTCAAATTTAA:6349

CSGH2 5679:GCTGTAGGAGTAAAGTCAG..AGCTTTCTCAAATTCTTCAATTAGTAAACTGTTGTTTAA:5736

ASGH2 5765:GTGTTATGCAAAGAACCCATTACAATATCAACATTTGTCAA...TTCCTTTCATGAAAAC:5821

. . .6790 . . .6800 . . .6810 . . .6820 . . .6830 . . .6840

ASGH1 6030:TTATAT..TCAACATTCGTGACA....CACAGTGGACGTGAATTTCACT...TTCAGGTT:6080

CSGH1 6350:TTATATATTCAACATTCGTGACA....CACAGTGGATGTGAAGCTCGCT...TTCAGGTT:6402

CSGH2 5737:CATTGACATTTACAGCTATATCAACTGTTCGGTGGTCAAACTATTTTGT...TGGATGCT:5793

ASGH2 5822:CTTTACAAGTCACAATTTCAGGAAGATGGTTGTGGAAGCTACATTTACCACATTCAGAAT:5881

. . .6850 . . .6860 . . .6870 . . .6880 . . .6890 . . .6900

ASGH1 6081:TT.GGCAATGG..ATTTTTGCGATGCTGAT...GATGTCATAGTGATAGCATCGGTGCAT:6134

CSGH1 6403:TTTGGCAATGG..ATTTTTGCGGCGCTGAT...GATGTCAGAGCGACAGCATCGGTGCAT:6457

CSGH2 5794:CCAAGTGAGGACAATATTGGACGGAATGGA...GATGCTAGATAGATACAACGAGAGGAC:5850

ASGH2 5882:TATGTCCCTGTAATTGAGGATGATTTCCACTCAGTTTTCGATTCACCCGTGTCAATTGAA:5941

. . .6910 . . .6920 . . .6930 . . .6940 . . .6950 . . .6960

ASGH1 6135:CATGACTAACAAG.TTCCATCCATGAAGTATAATGAACATTTTAATGTTATA..TGAATG:6191

CSGH1 6458:CATGACTAACAAG.TTCCATCCATGAAGTATAATGAACATTTTAATTTTAGA..TGAATG:6514

CSGH2 5851:GAGAAGGATGAGGGTTTCCGGAGTAGAGGAATATGGAAGGTGGATGGACAAACTTGAATC:5910

ASGH2 5942:GAAATTAGAGAGGCTCTGAATTCAATGAAAAAAGGGAAATCACCTGGTCCTGATGGCCTG:6001

. . .6970 . . .6980 . . .6990 . . .7000 . . .7010 . . .7020

ASGH1 6192:TGAGTTGAGAGTCTTAGA.CGTACCAGAA..TCTTAGATT.TACAAAAGC..ACCACGCC:6245

CSGH1 6515:TGAGTTGAGAGACACAGG.CATACCAGAA..TCGTAGATT.TACAAAAGC..ACCACGCC:6568

CSGH2 5911:TGGCCCGGTGGTGACAGGTCGTGCAAGGG..CAGAAGCTGATGCAATGTTTGATTCTGTT:5968

ASGH2 6002:TCAGTTGAATTCTATAGACAGTTTTGGGAGTTATTAGAAGACCCTATTTTT.AATATGTT:6060

. . .7030 . . .7040 . . .7050 . . .7060 . . .7070 . . .7080

ASGH1 6246:ACCCCACATCAACTC..CCACCATATCTCAATCT...........ATGGACTTACTTTCA:6292

CSGH1 6569:ACCCCACATCAACTC..CCACCATATCTCAATCT...........ATGGACTTACCTTCA:6615

CSGH2 5969:GGCGCGAGTAAGGTTGGTAAGAATGACTGGACCACGGTAATAAAGAAGAATTGAGCTAAA:6028

ASGH2 6060:..TCAAGATTGCATTGAAAATGGGGAAATGGTCTCCACTATGAAACAGGGTCTTATTTCA:6118

. . .7090 . . .7100 . . .7110 . . .7120 . . .7130 . . .7140

ASGH1 6293:GTACTT..GTAGCTGTGTTATAACTTATCATTCATCATTAA.....TGTCAATGTCATGA:6345

CSGH1 6616:GTACTT..GTAGCTGTGTTATAACTTATCATTCATCAGTAA.....GGTCAATGCCATGA:6668

CSGH2 6029:AGAGCT..AAAGCTGAAAAATGAACAGCAGTTTCTGGTTGG.....AGTACCATTCATGA:6081

ASGH2 6119:CTGATTCCGAAGCCCGATAAAGACCCTTCTCTCATTGACAATTGGAGACCAATTACTTCA:6178

. . .7150 . . .7160 . . .7170 . . .7180 . . .7190 . . .7200

ASGH1 6346:CCAGTGGCGTACCACAGATTAGTGAGGCTCCCCTGCAAGGCACGAGCAAGTCCAATTGTA:6405

CSGH1 6669:CCAGTGGTGTACCACAGATTCGTGAGGCTCCCCTGCAAGGCACGAGCAAGTCCAATTGTA:6728

CSGH2 6082:ATAATAATGT...CTATTTTTGGGAGACCTGTTTGCGAT.TACGAAGATGGTAAAGGACA:6137

ASGH2 6179:TTAAATATTGATTACAAATTGATTGCTCTGGTTTATGCCAAAAGAAAGGAATAGGTACCA:6238

. . .7210 . . .7220 . . .7230 . . .7240 . . .7250 . . .7260

ASGH1 6406:AAGCCAGGGGACGGGAAATAG..AATGTGATGG..TAATATTCATTTTTTTTACCTCAGG:6461

CSGH1 6729:AAGCCAGGGGACGGGAAATAG..AATGTGATGG..TAATATTCAATTTTTA.ACCTCAGG:6783

CSGH2 6138:AATTGAGAAAGGCGGAGTCAGTCAGAGTGACCA..GGAGCGGTATGATGGTGATTTCATG:6195

ASGH2 6239:TTATAAATGAGACTCAAACAGGATTTATGAGGGACCGTCACATAAGCTCTAACATTCATT:6298

. . .7270 . . .7280 . . .7290 . . .7300 . . .7310 . . .7320

ASGH1 6461:.AACATTTTACCTT...GATTGCCAAAAGACAATTAGCCTACCAAACTCTCTGAAAATGG:6517

CSGH1 6783:.AACATTTGACCTT...GATTGCCAAAAGACAATTATCCTACCAACCTCTCTGAAAATGG:6839

CSGH2 6195:.TGTATCAAAAC.....AGCAAAAGGGAGACAGCTCTGTGGCTCTATAAACTGTCAACAT:6249

ASGH2 6299:TAGTCTTGGACCTTCTAGATTATTCAGATGCAATTGACTCAGTCTTATTTTTG.....GA:6353

. . .7330 . . .7340 . . .7350 . . .7360 . . .7370 . . .7380

ASGH1 6518:TTCGAGTGAAGGGACTTCAATGGTGAGATAAAACGATTTGATTATTTTACT.TTTCTCAG:6576

CSGH1 6840:TTCTAGTGAAGGGACTTCAATGGTGAGGTAAAACAATTTGATTATTTTACT.TTTCTCAG:6898

CSGH2 6250:ACGAAGTGGAGAGTTGTGATTTTCAAGAGAAGGGCACCGAAGAAAGGTGTCATCTCGCTG:6309

ASGH2 6354:CTTCTGTAAAGCCTTTGACACAATTGAACATGAATCTCTCTTTAGGTCACTTAACATTTT:6413

. . .7390 . . .7400 . . .7410 . . .7420 . . .7430 . . .7440

ASGH1 6577:GTCTTCGATCATGGGCGACTCCAAACCCTT.TGGTGGCCTCAACCAACT........TTA:6627

CSGH1 6899:GTCTTCGATCATGGGCGACTCCAAACCCTT.TGGTGGCCTCAACCAACT........TTA:6949

CSGH2 6310:GATATCGAGGCTTCACATCACTTAACCCTTATTGTGAAAGGAAAGAAGGAGGAGAAGTCT:6369

ASGH2 6414:CACCGAAGCCAAAGAGTTTATCAAAGTCAT.TTGCATGTTTTACGAAGGTATA...AATA:6469

. . .7450 . . .7460 . . .7470 . . .7480 . . .7490 . . .7500

ASGH1 6628:ACCAGATGAAG.GTATGTCCCAGCACTTGCAATTTCATTGGTTACA...GAACTGTAAA.:6682

CSGH1 6950:ACCAGATGAAG.GTGTGTCCCAGCACTTGCAATTTCATTGGTTACA...GAACTCCAAAT:7005

CSGH2 6370:GTTCGATGAAGAGTCTCTCCTGACCCATGTAAAGCTGT..GATACAT..GAGATGTGCGG:6425

ASGH2 6470:GTTCTGTGTTA.CTAAACCTTAATACATCCAAAAGATTCAGTATCAACAGAAGAGTATGA:6528

. . .7510 . . .7520 . . .7530 . . .7540 . . .7550 . . .7560

ASGH1 6683:CACCGCCCCGTGCTCGAGC..TCATTGGTTGTGTTGCGTACAGAAAATCTGCTGTAG.GA:6739

CSGH1 7006:CACATACACATGGTT.AGC..AGAT..GTTAATGCGAGTGTAGCAAAATGCTTGT...GC:7057

CSGH2 6426:TGCCAATGTATGTACAAAA..GTAACAGCAATGTCATGCAT.GCAAATCTTTGGCCACGT:6482

ASGH2 6529:CGGGGATGCCCAATTTCGCCATTTTTATTCATTTTGGTTGTGGAAATTCTATCTCTACAT:6588
