# Supplementary figures and images for: Isolation, characterization and comparison of Atlantic and Chinook salmon growth hormone 1 and 2
Source: BMC Genomics. 2008 Nov 3;9:522. doi: 10.1186/1471-2164-9-522 (PMC2584663; doi:10.1186/1471-2164-9-522)

## Slide 1
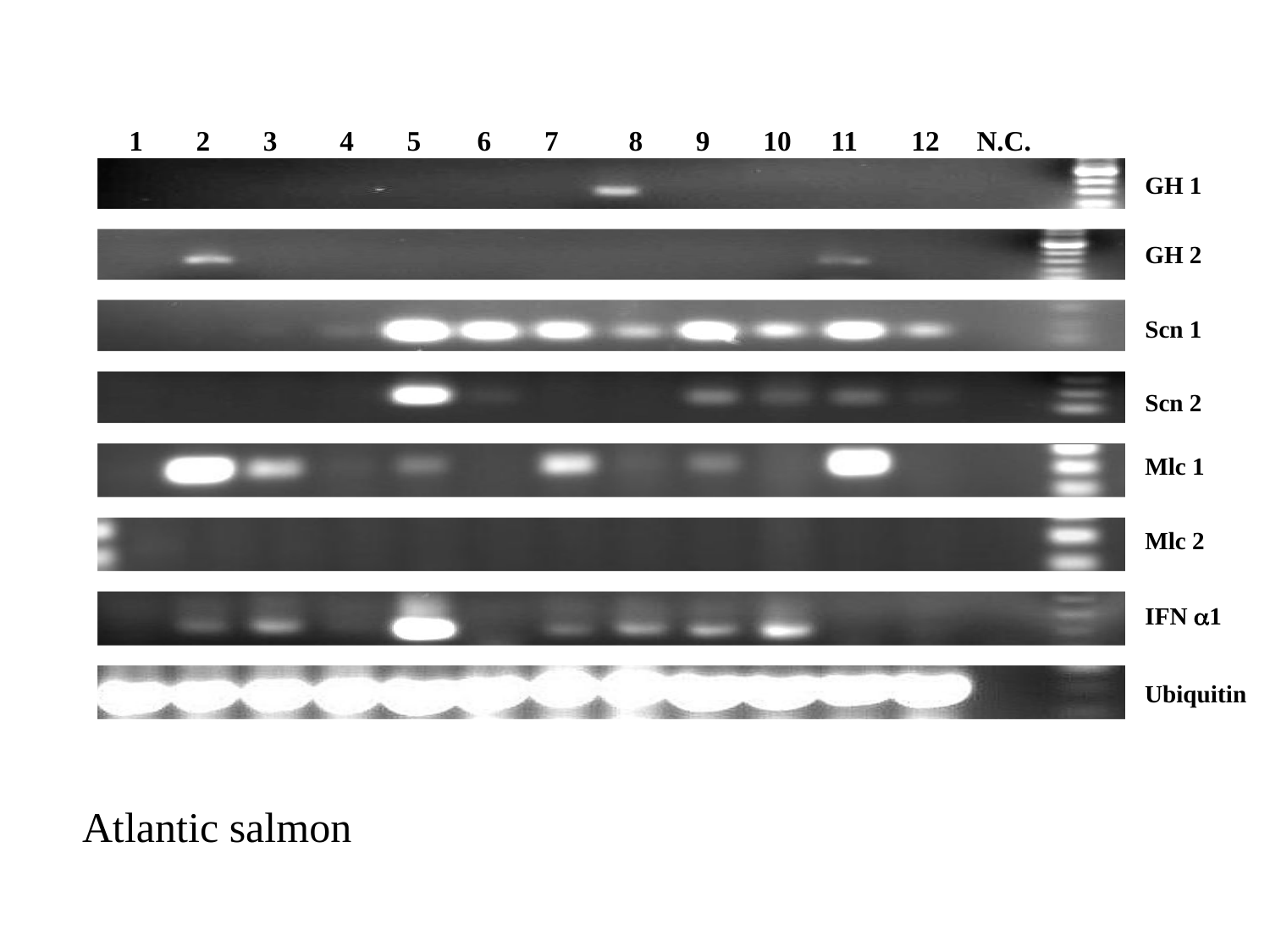

1
2
3
4
5
6
7
8
9
10
11
12
N.C.
GH 1
GH 2
Scn 1
Scn 2
Mlc 1
Mlc 2
IFN α1
Ubiquitin
Atlantic salmon

Supplement: Additional file 2 — Reverse transcriptase PCR validation and cDNA expression profiles in twelve different tissues. Reverse transcriptase PCR validation and cDNA expression profiles in twelve different tissues: 1: kidney, 2: muscle, 3: skin, 4: gut, 5: gill, 6: spleen, 7: brain, 8: heart, 9: testis, 10: liver, 11: eye and 12: pyloric caecum. The integrity of each cDNA used was confirmed by control PCRs using ubiquitin primer set. For each gene-specific PCR experiment, a negative control with no template (NC) was included. Abbreviations for gene names are as follows: GH: growth hormone; Scn: skeletal muscle sodium channel alpha subunit; Mlc: myosin alkali light chain; IFN: interferon. The strongest marker band indicates a fragment length of 500 bp. [file 1471-2164-9-522-S2.ppt]

## Slide 1
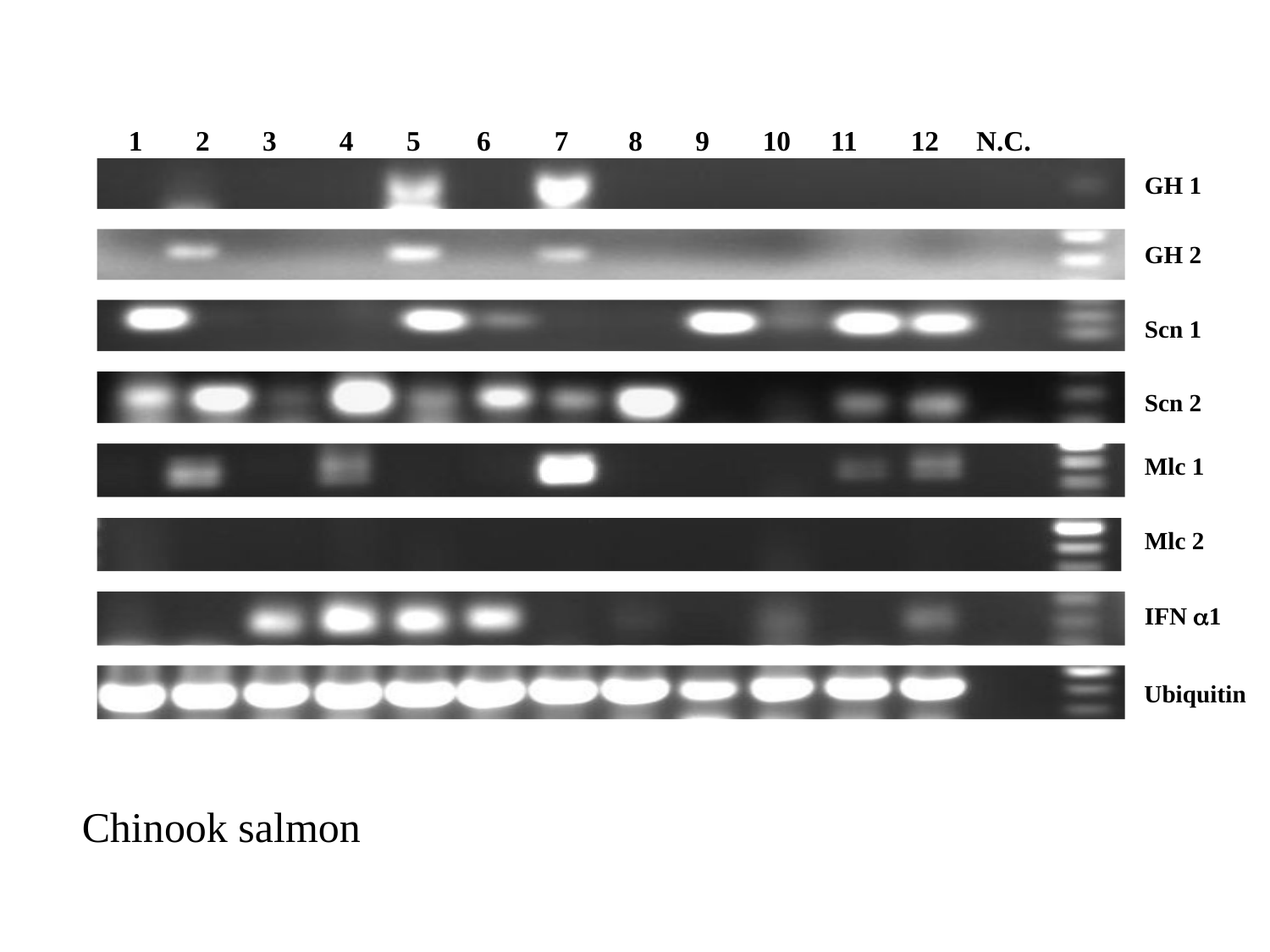

1
2
3
4
5
6
7
8
9
10
11
12
N.C.
GH 1
GH 2
Scn 1
Scn 2
Mlc 1
Mlc 2
IFN α1
Ubiquitin
Chinook salmon

Supplement: Additional file 3 — Reverse transcriptase PCR validation and cDNA expression profiles in twelve different tissues. Reverse transcriptase PCR validation and cDNA expression profiles in twelve different tissues: 1: kidney, 2: muscle, 3: skin, 4: gut, 5: gill, 6: spleen, 7: brain, 8: heart, 9: testis, 10: liver, 11: eye and 12: pyloric caecum. The integrity of each cDNA used was confirmed by control PCRs using ubiquitin primer set. For each gene-specific PCR experiment, a negative control with no template (NC) was included. Abbreviations for gene names are as follows: GH: growth hormone; Scn: skeletal muscle sodium channel alpha subunit; Mlc: myosin alkali light chain; IFN: interferon. The strongest marker band indicates a fragment length of 500 bp. [file 1471-2164-9-522-S3.ppt]
